# Assessment of Prevention Research Measuring Leading Risk Factors and Causes of Mortality and Disability Supported by the US National Institutes of Health

**DOI:** 10.1001/jamanetworkopen.2019.14718

**Published:** 2019-11-08

**Authors:** Ashley J. Vargas, Sheri D. Schully, Jennifer Villani, Luis Ganoza Caballero, David M. Murray

**Affiliations:** 1 Office of Disease Prevention, National Institutes of Health, North Bethesda, Maryland; 2 Scientific Consulting Group, Inc, Gaithersburg, Maryland

## Abstract

**Importance:**

No studies to date have examined support by the National Institutes of Health (NIH) for primary and secondary prevention research in humans and related methods research that measures the leading risk factors or causes of death or disability as outcomes or exposures.

**Objective:**

To characterize NIH support for such research.

**Design and Setting:**

This serial cross-sectional study randomly sampled NIH grants and cooperative agreements funded during fiscal years 2012 through 2017. For awards with multiple subprojects, each was treated as a separate project. Study characteristics, outcomes, and exposures were coded from October 2015 through February 2019. Analyses weighted to reflect the sampling scheme were completed in March through June 2019. Using 2017 data from the Centers for Disease Control and Prevention and 2016 data from the Global Burden of Disease project, the leading risk factors and causes of death and disability in the United States were identified.

**Main Outcomes and Measures:**

The main outcome was the percentage of the NIH prevention research portfolio measuring a leading risk factor or cause of death or disability as an outcome or exposure.

**Results:**

A total of 11 082 research projects were coded. Only 25.9% (95% CI, 24.0%-27.8%) of prevention research projects measured a leading cause of death as an outcome or exposure, although these leading causes were associated with 74.0% of US mortality. Only 34.0% (95% CI, 32.2%-35.9%) measured a leading risk factor for death, although these risk factors were associated with 57.3% of mortality. Only 31.4% (95% CI, 29.6%-33.3%) measured a leading risk factor for disability-adjusted life-years lost, although these risk factors were associated with 42.1% of disability-adjusted life-years lost. Relatively few projects included a randomized clinical trial (24.6%; 95% CI, 22.5%-26.9%) or involved more than 1 leading cause (3.3%; 95% CI, 2.6%-4.1%) or risk factor (8.8%; 95% CI, 7.9%-9.8%).

**Conclusions and Relevance:**

In this cross-sectional study, the leading risk factors and causes of death and disability were underrepresented in the NIH prevention research portfolio relative to their burden. Because so much is already known about these risk factors and causes, and because randomized interventions play such a vital role in the development of clinical and public health guidelines, it appears that greater attention should be given to develop and test interventions that address these risk factors and causes, addressing multiple risk factors or causes when possible.

## Key Points

**Question:**

Does the National Institutes of Health fund prevention research of the leading risk factors and causes of death and disability proportionate to their burden?

**Findings:**

In this cross-sectional study of 11 082 grants and cooperative agreements from the National Institutes of Health, the level of prevention research measuring the leading risk factors and causes of death as exposures or outcomes fell well below their US burden of mortality and disability.

**Meaning:**

Results suggest that greater attention should be given to develop and test preventive interventions for the leading risk factors and causes of death and disability in the United States, addressing multiple risk factors or causes when possible.

## Teaser

This cross-sectional study characterizes National Institutes of Health (NIH) support for primary and secondary prevention research in humans and related methods research of the leading risk factors or causes of death or disability as outcomes or exposures.

## Introduction

The United States faces numerous public health challenges, from expanding waistlines to opioid overdoses. To develop evidence-based interventions, the United States relies on the National Institutes of Health (NIH) to support cutting-edge biomedical research. The NIH fulfills its mission to enhance health, lengthen life, and reduce illness and disability by distributing more than 80% of its budget to external researchers to support peer-reviewed biomedical research. The burden of disease in the United States is one of the factors the NIH considers in allocating its resources.^1^

Previous studies^2,3,4,5,6^ have examined the association between NIH funding and burden of disease measures, such as mortality. Gross et al^2^ reported that disease-specific NIH funding levels for 1996 had only a weak association with mortality rates. Gillum et al^3^ found no association more than 10 years later. Those analyses preceded the development of a consistent method to estimate disease-specific funding levels across the NIH. That method, called the Research, Condition, and Disease Categorization system,^4^ was introduced in 2008 and used by Sampat et al,^5^ who observed a positive association between US burden of disease and the level of NIH funding. A more recent study^6^ reported positive associations between NIH funding and deaths and disability-adjusted life-years (DALYs) in the United States and globally.

These studies focused on the full spectrum of research at the NIH, including basic, preclinical, clinical, and prevention. Each of these areas is important, and developments in one area often lead to progress in another. Because prevention research can more rapidly affect public health than basic or preclinical research, this study focuses on primary and secondary prevention research in humans, together with related methods research. Hereafter, we refer to such research simply as prevention research. We define the NIH prevention research portfolio as grants and cooperative agreements that include prevention research and were awarded during fiscal years 2012 through 2017.^7,8^ The NIH prevention research portfolio accounts for 16.7% of all NIH research projects funded during these fiscal years.^7^

We examine support for prevention research as it relates to the diseases and risk factors that make the largest contribution to US mortality and disability. We used 2016 data on risk factors from the Global Burden of Disease (GBD) Study^9^ in conjunction with 2017 data from the Centers for Disease Control and Prevention (CDC) on the leading causes of death.^10^

## Methods

### Portfolio Analysis

We developed a taxonomy, a manual coding process, and a statistical sampling approach for a serial cross-sectional study to characterize the NIH portfolio of prevention research. Details are provided elsewhere, and only a brief description is provided here.^7,8^ All work was conducted in Bethesda, Maryland, from October 2015 through February 2019. The NIH did not require institutional review board approval because this analysis included only administrative data and no data on humans. This study followed the reporting requirements of the Strengthening the Reporting of Observational Studies in Epidemiology (STROBE) Statement.^11^

New research projects were defined as type 1 (new), 2 (renewal), or 9 (change of institute at the time of renewal) projects^12^; for awards with multiple subprojects, each subproject was treated as a separate project. We selected 12 activity codes (R01, R03, R21, R43, R44, R56, U01, U19, U54, UM1, P01, and P50) that supported 91.7% of all new research awards made using grants and cooperative agreements during fiscal years 2012 through 2017.^7^ We identified all new research projects funded using these activity codes during this period (64 744 projects from 58 104 awards).

In late 2017, machine learning was applied to this set of research projects to identify those that were likely to include prevention research.^7,8^ During 2018, we used a stratified sampling procedure to sample projects from this set; the strata were type (1, 2, and 9), activity code (the 12 codes noted above), fiscal year (2012-2017), and machine learning prediction (prevention or not prevention). For most types and activity codes, we selected 50% of the projects predicted to include prevention and 5% of the projects predicted not to include prevention in each fiscal year. Type 1 R01s were the most common awards, and we selected 100% of the projects intending to include prevention and 5% of the projects intending not to include prevention in each fiscal year. In all, we coded 71.3% (8346 of 11 713) of the research projects intending to include prevention research and 5.2% (2736 of 53 031) of the research projects intending not to include prevention research. The sampling fractions were selected based on considerations for budget and precision.

Coding data used for this analysis was originally generated between October 2015 and February 2019. Specifically, a group of 3 research analysts read the title, abstract, and public health relevance of each sampled research project and manually coded that project according to a list of 128 topics defined in the Office of Disease Prevention (ODP) Taxonomy Protocol (eMethods in the Supplement). These topics were grouped into the following 6 categories: study rationale, exposure, outcome, population focus, study design, and prevention research category. Coders selected all topics within each category that applied to a given project. After coding each project individually, the research analysts discussed their coding to reach a set of consensus codes for each project. A team of NIH staff scientists (A.J.V., S.D.S., J.V., and L.G.C.) reviewed a random sample of 10% to 20% of each week’s coded projects for quality control.

### Aligning Definitions

The 10 leading causes of death and the 10 leading risk factors for death and DALYs lost in the United States were identified from the CDC’s Center for Vital Statistics^10^ and a recent GBD report,^9^ respectively; all are listed in the Table. Many of the top 10 risk factors for death and DALYs lost were the same (7 of 10), leaving 10 unique causes and 13 unique risk factors. The definitions of these 23 unique risk factors and causes were compared with the definitions in the ODP Taxonomy Protocol (eMethods in the Supplement). Sixteen had definitions that aligned well; for these, the ODP Taxonomy Protocol–based manual coding results were used to identify projects in which 1 or more of these risk factors or causes were measured as an exposure or an outcome. For the remaining 7 risk factors and causes, 2 research assistants performed additional coding that was reviewed by an NIH staff scientist (A.J.V., S.D.S., J.V., and L.G.C.) in 2019. The ODP definitions for heart disease, lung disease, and kidney disease were broader than the CDC definitions, and the ODP definition of kidney disease was broader than the GBD definition of impaired kidney function; projects that did not meet the CDC or GBD definition were recoded. Projects focusing on high fasting plasma glucose levels were identified if they were coded for diabetes using the ODP definition or if they met the GBD definition after manually coding projects with specific key words (*glycemia*, *glycaemia*, *glucose*, *blood glucose*, *hemoglobin A_1c_*, and *HbA_1c_*) in their title, abstract, or public health relevance. Projects focusing on air pollution were identified if they were coded for chemical and/or toxin using the ODP definition or if they met the GBD definition after we manually coded projects containing specific key words (*pollution*, *particulate*, *ppm* [parts per million], and *PM_2.5_* [<2.5 μm in diameter]) in their title, abstract, or public health relevance. All projects coded as blood pressure or cholesterol using the ODP definition under high systolic blood pressure or high total cholesterol level were retained because often researchers did not state the exact type of blood pressure or cholesterol they were measuring. The GBD does not include low physical activity measures in children owing to feasibility, but the ODP definition includes all prevention projects studying low physical activity regardless of the age group, and the ODP definition was used. Projects focusing on occupational risks were identified if they met the GBD definition after manually coding projects with specific key words (job OR worker OR labor* OR employ* OR occupation*) and key words from the GBD definition in their title, abstract, or public health relevance. A project was considered to address a leading risk factor or cause of death if any aim measured a leading risk factor or a cause of death as an exposure or outcome.

**Table.** Prevention Research Measuring the Leading Causes or Risk Factors for Death or Disability

Leading Causes or Risk Factors for Death or Lost DALYs	NIH Prevention Research Portfolio		% of Attributable Deaths
	Projects, % (95% CI)	Dollars, % (95% CI)	
Any top 10 leading cause of death^a^	25.9 (24.0-27.8)	28.2 (24.8-31.5)	74.0
Heart disease	4.2 (3.3-5.2)	4.8 (3.2-6.4)	23.0
Cancer	11.9 (10.5-13.4)	11.3 (9.2-13.4)	21.3
Accidents	1.7 (1.2-2.4)	1.7 (1.1-2.4)	6.0
Chronic lower respiratory disease	1.8 (1.4-2.3)	2.0 (1.3-2.7)	5.7
Stroke	2.7 (2.2-3.4)	3.3 (2.3-4.3)	5.2
Alzheimer disease	2.0 (1.4-2.6)	3.2 (1.9-4.6)	4.3
Diabetes	3.6 (3.0-4.2)	4.5 (3.4-5.6)	3.0
Influenza or pneumonia	0.5 (0.2-1.0)	0.7 (0.1-1.3)	2.0
Kidney disease	1.4 (0.9-2.2)	1.4 (0.8-2.0)	1.8
Suicide	0.7 (0.5-0.9)	0.7 (0.5-0.9)	1.7
Any top 10 risk factor for death^b^	34.0 (32.2-35.9)	32.5 (28.9-36.2)	57.3
Dietary risk	7.8 (7.0-8.8)	6.7 (5.7-7.7)	19.1
Tobacco use	6.6 (5.8-7.6)	5.4 (4.5-6.3)	17.8
High systolic blood pressure	2.7 (2.2-3.3)	3.1 (2.3-3.9)	17.4
High body mass index	5.3 (4.7-6.0)	6.5 (3.7-9.4)	13.9
High fasting plasma glucose level	4.6 (3.9-5.4)	6.6 (3.7-9.6)	13.6
High total cholesterol level	1.8 (1.4-2.3)	2.0 (1.3-2.6)	8.4
Impaired kidney function	1.6 (1.0-2.3)	1.6 (1.0-2.3)	6.3
Alcohol and/or drug use	11.2 (10.2-12.4)	10.2 (8.5-11.8)	5.6
Air pollution	1.4 (1.1-1.6)	1.4 (0.9-1.8)	3.8
Low physical activity	5.0 (4.4-5.7)	4.3 (3.7-4.8)	3.3
Any top 10 risk factor for lost DALYs^b^	31.4 (29.6-33.3)	30.3 (26.6-33.9)	42.1
High body mass index	5.3 (4.7-6.0)	6.5 (3.7-9.4)	11.6
Tobacco use	6.6 (5.8-7.6)	5.4 (4.5-6.3)	11.1
Dietary risk	7.8 (7.0-8.8)	6.7 (5.7-7.7)	10.4
High fasting plasma glucose level	4.6 (3.9-5.4)	6.6 (3.7-9.6)	9.7
High systolic blood pressure	2.7 (2.2-3.3)	3.1 (2.3-3.9)	8.0
Drug use	7.3 (6.4-8.2)	7.6 (6.0-9.2)	6.5
Alcohol use	5.6 (4.9-6.4)	4.1 (3.6-4.7)	4.2
High LDL cholesterol level	1.8 (1.4-2.3)	2.0 (1.3-2.6)	4.0
Impaired kidney function	1.6 (1.0-2.3)	1.6 (1.0-2.3)	3.1
Occupational risks	0.3 (0.1-0.4)	0.2 (0.1-0.3)	2.5

Abbreviations: DALY, disability-adjusted life-years; LDL, low-density lipoprotein; NIH, National Institutes of Health.

^a^ The top 10 leading causes of death in the United States for 2017 and percentage of attributable deaths are from the Centers for Disease Control and Prevention.^10^

^b^ The top 10 leading risk factors for death or loss of DALYs in the United States for 2016 and percentage of attributable deaths are from the study by Mokdad et al^9^ and as part of the Global Burden of Disease Study.

### Statistical Analysis

Analyses weighted to reflect the sampling scheme were completed March through June 2019. The 11 082 coded projects were weighted to represent the entire population of 64 744 new research projects awarded by NIH during fiscal years 2012 through 2017 using the 12 selected activity codes. Weights were assigned based on the stratified sampling scheme; for example, if 37 of 127 projects in a given activity code, type, fiscal year, and prediction combination were coded, each of those projects was assigned a weight of 3.7. The data were analyzed using the SVYSET function in Stata/SE, version 15 (StataCorp LLC) in early 2019. A finite population correction was used in conjunction with the standard settings of SVYSET. Proportions and their 95% CIs were calculated using SVYSET. Two-sided *P* values for trends were calculated using logistic regression within SVYSET, with *P* < .05 indicating statistical significance. All analyses were unadjusted.

## Results

### Levels and Trends of Prevention Research Involving the Leading Risk Factors or Causes of Death and Disability

For fiscal years 2012 through 2017, we estimated that 51.4% (95% CI, 49.3%-53.6%) of NIH-funded prevention research projects and 50.8% (95% CI, 46.2%-55.3%) of prevention research dollars measured a leading risk factor or cause of death as an exposure or as an outcome. We estimated that 31.4% (95% CI, 29.6%-33.3%) of prevention research projects and 30.3% (95% CI, 26.6%-33.9%) of prevention research dollars measured a leading risk factor for DALYs lost (Table). Because the results for prevention research dollars closely followed the results for prevention research projects (Table), the remainder of this report will present results in terms of percentage of prevention research projects.

Overall, 25.9% (95% CI, 24.0%-27.8%) of projects measured any leading cause of death, and this 95% CI did not include the total burden of these diseases on mortality (74.0%) (Table). A total of 34.0% (95% CI, 32.2%-35.9%) of projects measured any leading risk factor for death, and this 95% CI did not include the total burden of these risk factors on mortality (57.3%) (Table). A total of 31.4% (95% CI, 29.6%-33.3%) of projects measured any leading risk factor for DALYs lost, and this 95% CI did not include the burden of these risk factors on disability (42.1%) (Table). The fractions of the NIH prevention research portfolio that measured other exposures and outcomes are provided in eTable 1 in the Supplement.

The 95% CIs for all but 2 of the leading causes of death were lower than the CDC percentage of deaths associated with those causes (Table). Overall, the leading causes of death were more often measured as an outcome (25.2%; 95% CI, 23.3%-27.1%) than an exposure (2.0%; 95% CI, 1.7%-2.4%) (eTable 2 in the Supplement).

Similarly, the 95% CIs for all but 2 of the leading risk factors for death were lower than the CDC percentage of deaths associated with those risk factors (Table). Overall, the leading risk factors for death were more often measured as exposures (15.0%; 95% CI, 13.8%-16.2%) than were the leading causes of death; however, in general, risk factors were more frequently measured as outcomes (26.3%; 95% CI, 24.7%-28.0%) than as exposures (eTable 3 in the Supplement).

**Figure 1.**
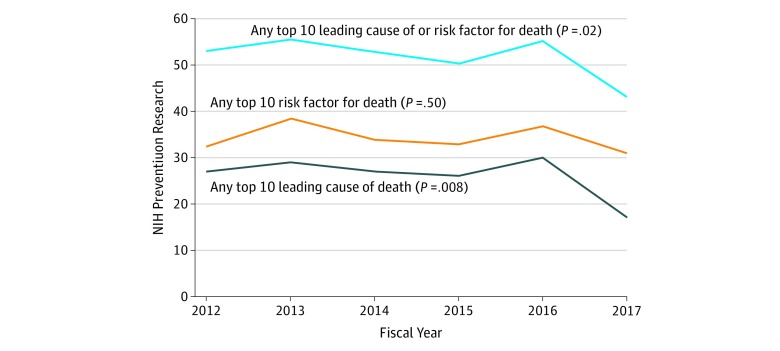
Trends in Prevention Research Measuring the Leading Risk Factors or Causes of Death The percentage of prevention research involving leading risk factors or causes of death in the United States was stable from fiscal years 2012 through 2016. A large decrease in the amount of National Institutes of Health–supported cancer prevention research in 2017 (eFigure 1 in the Supplement) drove the significant decrease in the percentage of prevention research involving the leading causes of death (*P* = .008 for trend) and the combined leading risk factors and/or causes of death (*P* = .02 for trend). No other leading risk factors or causes of death were observed to have significant changes over time (more details are given in eFigure 1 and eFigure 2 in the Supplement). If any aim of a prevention research project measured a leading risk factor or cause of death as an exposure or outcome for a hypothesis, then the prevention research project was considered to address those leading risk factors or causes.

Prevention research that measured the leading risk factors or causes of death as an exposure or an outcome was relatively stable during fiscal years 2012 through 2017, although a decrease was noted in 2017 (Figure 1) (2012, 53.0% [95% CI, 47.6%-58.4%]; 2013, 55.3% [95% CI, 49.5%-61.0%]; 2014, 52.8% [95% CI, 47.3%-58.2%]; 2015, 50.2% [95% CI, 45.1%-55.4%]; 2016, 55.2% [95% CI, 50.4%-59.9%]; 2017, 43.0% [95% CI, 38.1%-47.9%]; *P* = .02), driven by a decrease in prevention research projects that measured cancer as an exposure or outcome (eFigure 1 in the Supplement) (2012, 14.1% [95% CI, 10.3%-19.0%]; 2013, 14.2% [95% CI, 10.4%-19.1%]; 2014, 13.9% [95% CI, 10.3%-18.6%]; 2015, 12.0% [95% CI, 9.6%-14.9%]; 2016, 11.6% [95% CI, 9.2%-14.5%]; 2017, 5.7% [95% CI, 3.9%-8.2%]; *P* < .001 for cancer). No other major trends associated with the leading risk factors or causes of death were observed (eFigure 2 in the Supplement).

### Prevention Research Involving Multiple Risk Factors or Causes of Death

Only 3.3% (95% CI, 2.6%-4.1%) of prevention research projects measured more than 1 leading cause of death as an exposure or outcome. Only 8.8% (95% CI, 7.9%-9.8%) of prevention research projects measured more than 1 leading risk factor for death as an exposure or outcome.

### Prevention Research Involving Leading Risk Factors or Causes of Death by Population Focus

**Figure 2.**
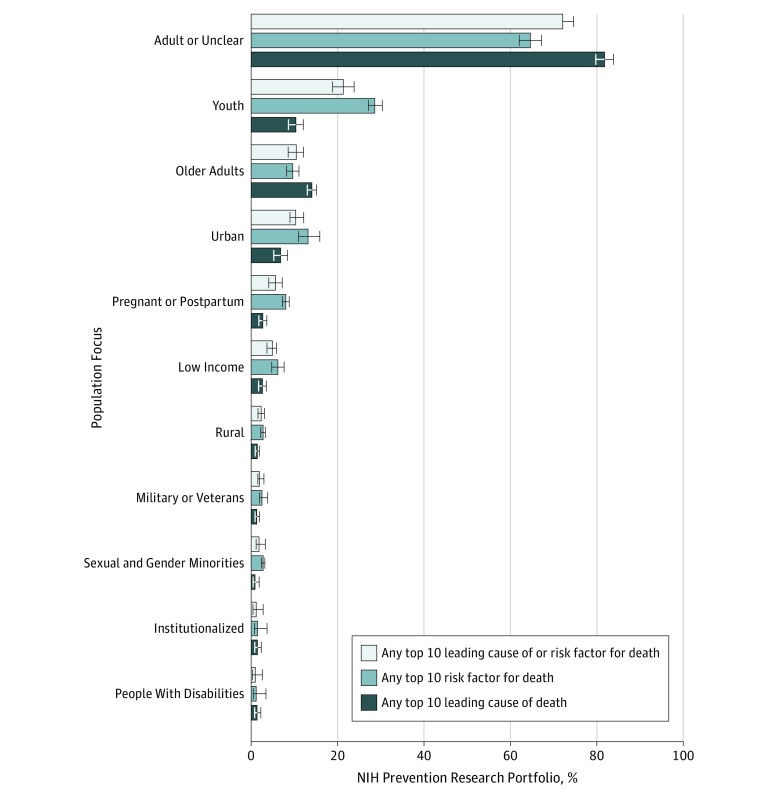
Prevention Research Measuring the Leading Risk Factors or Causes of Death by Population Studied Most prevention research involving leading risk factors or causes of death identified a general, adult, or nonspecific study population (72.0%; 95% CI, 69.9%-74.1%). Risk factors were more often studied in youth (28.7%; 95% CI, 26.5%-30.9%) than were leading causes of death (10.3%; 95% CI, 8.8%-12.0%). Conversely, leading causes were often studied among older adults (14.1%; 95% CI, 11.8%-16.7%) than were risk factors (9.6%; 95% CI, 8.1%-11.4%). If any aim of a prevention research project measured a leading risk factor or cause of death as an exposure or an outcome for a hypothesis, then the prevention research project was considered to address those leading risk factors or causes. All populations specified as a focus for a prevention research project were coded for the portfolio analysis. Sexual and gender minorities includes lesbian, gay, bisexual, transgender, and intergender populations and other sexual minorities, as well as men who have sex with men. Error bars indicate 95% CIs.

Most prevention research projects measuring leading risk factors or causes of death as an exposure or outcome focused on the general population (adult or unspecified) (72.0%; 95% CI, 69.9%-74.1%) (Figure 2). When youth were the focus, risk factors (28.7%; 95% CI, 26.5%-30.9%) were measured more often than leading causes of death (10.3%; 95% CI, 8.8%-12.0%), and when older adults were the focus, leading causes of death (14.1%; 95% CI, 11.8%-16.7%) were measured more often than risk factors (9.6%; 95% CI, 8.1%-11.4%).

### Prevention Research Involving Leading Risk Factors or Causes of Death by Study Design

**Figure 3.**
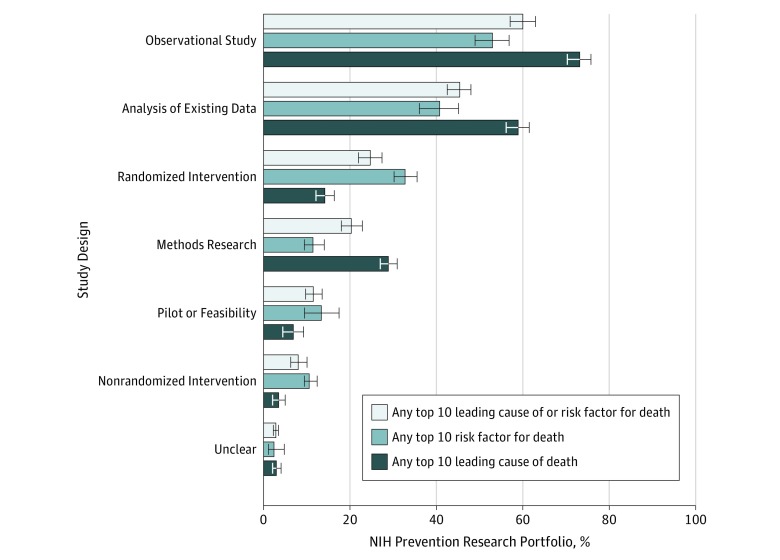
Prevention Research Measuring the Leading Risk Factors or Causes of Death by Study Design Most prevention research involving leading risk factors or causes of death included an observational study design (60.0%; 95% CI, 57.2%-62.7%). Randomized clinical trials evaluating interventions were included more often in prevention research involving risk factors (32.6%; 95% CI, 30.0%-35.4%) as opposed to the leading causes of death (14.1%; 95% CI, 11.6%-17.0%). Methods research (including biomarker development) was more commonly observed in prevention research projects involving the leading causes of death (13.3%; 95% CI, 11.0%-15.9%) than risk factors (6.7%; 95% CI, 4.8%-9.3%). If any aim of a prevention research project measured a leading risk factor or cause of death as an exposure or an outcome for a hypothesis, then the prevention research project was considered to address those leading risk factors or causes. All study designs for a prevention research project were coded for the portfolio analysis. Error bars indicate 95% CIs.

Many prevention research projects measuring the leading risk factors or causes of death as an exposure or outcome included an observational design (60.0%; 95% CI, 57.2%-62.7%) or an analysis of existing data (45.2%; 95% CI, 42.6%-47.9%) (Figure 3). Fewer projects included a randomized clinical trial to evaluate an intervention (24.6%; 95% CI, 22.5%-26.9%). In randomized interventions, risk factors were more often measured (32.6%; 95% CI, 30.0%-35.4%) than causes of death (14.1%; 95% CI, 11.6%-17.0%).

### Prevention Research on Leading Risk Factors and/or Causes of Death by Type of Prevention

**Figure 4.**
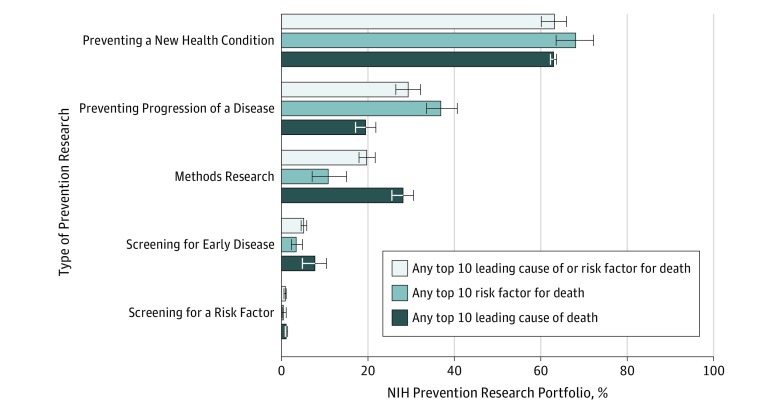
Prevention Research Measuring the Leading Risk Factors or Causes of Death by Type of Prevention Research Most prevention research involving a leading risk factor or cause of death included at least 1 aim to prevent a new health condition (63.2%; 95% CI, 60.3%-65.9%). Preventing progression of a disease was observed more often in prevention research involving risk factors (36.9%; 95% CI, 34.1%-39.9%) than leading causes of death (19.4%; 95% CI, 16.1%-23.2%). Methods research (including biomarker development) was observed more often among prevention research involving leading causes of death (28.0%; 95% CI, 24.2%-32.3%) than risk factors (10.8%; 95% CI, 9.1%-12.9%). If any aim of a prevention research project measured a leading risk factor or cause of death as an exposure or outcome for a hypothesis, then the prevention research project was considered to address those leading risk factors or causes. All types of prevention research specified for a prevention research project were coded for the portfolio analysis. Error bars indicate 95% CIs.

Leading risk factors and/or causes of death were most often measured in the context of preventing a new health condition (63.2%; 95% CI, 60.3%-65.9%) and least often measured in the context of screening for a risk factor (0.8%; 95% CI, 0.05%-0.11%) (Figure 4). Of interest, 36.9% (95% CI, 34.1%-39.9%) of prevention research projects measuring risk factors evaluated disease progression as opposed to only 19.4% (95% CI, 16.1%-23.2%) of projects measuring causes of death.

### Prevention Research Involving Leading Risk Factors and/or Causes of Death in the Context of the Larger Research Portfolio

Because only 16.7% of all NIH research projects include prevention research,^7^ only 34.0% of those projects addressed 1 of the leading risk factors for death, and only 24.6% of those projects evaluated an intervention in a randomized clinical trial, we estimate that only 1.9% (0.167 × 0.340 × 0.246 = 0.019) of the NIH research portfolio of grants and cooperative agreements supported prevention research that included a randomized intervention to address a leading risk factor. By similar calculation (0.167 × 0.259 × 0.246 = 0.011), we estimate that only 1.1% of the NIH research portfolio of grants and cooperative agreements supported prevention research that included a randomized intervention to address a leading cause of death, and only 0.3% and 0.2% supported prevention research to address screening for early disease in which a leading risk factor or cause of death was measured.

## Discussion

These results suggest that the leading risk factors and causes of death and disability are underrepresented in prevention research supported by the NIH relative to their contribution to mortality and disability in the United States. This was true in aggregate and for most individual causes and risk factors and for the proportion of prevention research projects and for dollars. Herein we draw attention to a few key findings.

There is considerable overlap among the risk factors for the leading causes of death (eg, cancer, cardiovascular disease, stroke, and diabetes^13,14,15^), and many risk factors co-occur in the same individuals (eg, tobacco use, alcohol and/or other drug use, low physical activity, obesity, and poor dietary habits^16,17^). Even so, only 3.7% of prevention research projects measured more than 1 leading risk factor or cause of death or disability as an exposure or outcome. Measuring more than 1 risk factor or cause of death or disability as an exposure or outcome would make more efficient use of existing resources and help the NIH meet its strategic objective to enhance scientific stewardship.^1^ Investigators could use these findings in future applications to justify studies that address multiple risk factors or causes of death. The NIH could consider changes to policies or practices that would encourage this approach.

We found that randomized clinical trials were less common than observational studies or analyses of existing data in prevention research measuring the leading risk factors and/or causes of death as exposures or outcomes. Because so much of the variability in US county-level life expectancy^18^ and mortality^19^ is associated with the leading risk factors and causes of death and disability, we believe the nation would be well served if the NIH had a more robust portfolio of prevention research that developed and tested interventions to address those risk factors and causes. Such trials would provide the evidence required by the US Preventive Services Task Force and the Community Preventive Services Task Force, among others, as they develop clinical and public health guidelines to improve the health of all US individuals. Investigators are encouraged to use these findings in future applications to justify studies to develop and test interventions to address these risk factors and causes. Again, the NIH could consider changes to policies and procedures that would encourage this approach.

There was little research on screening for early disease in which a leading risk factor or cause of death was measured as an outcome or an exposure. Less than 1% of NIH grants and cooperative agreements funded prevention research focused on screening for early disease in which a leading risk factor or cause of death was measured as an outcome or an exposure. These proportions are very low, given the number of insufficient evidence statements issued by the US Preventive Services Task Force related to screening for early disease.^20^

If only 51.4% of NIH-funded prevention research projects measured a leading risk factor or cause of death as an exposure or as an outcome, what was the focus of the remaining 48.6% of prevention research projects? Those exposures and outcomes are listed in eTable 1 in the Supplement. The list of topics is long and varied, and all deserve some level of prevention research support. This is one of the challenges for the NIH, which has many worthy targets and a limited supply of resources. One of the judgments that NIH institutes and centers must make is how to balance resources among the many activities they fund. The purpose of this study was to clarify how much of the NIH’s research portfolio is used to support prevention research that measures the leading risk factors and causes of death and disability in the US decision makers, and the public can judge whether sufficient resources are being targeted to these risk factors and causes.

### Limitations

This study has some limitations. This portfolio analysis was restricted to grants and cooperative agreements; some NIH institutes and centers use contracts and their intramural research program to support prevention research, and that work was not represented herein. The intramural program represented 10.9% of the NIH budget for the fiscal year 2017, and contracts represented 5.9%, and most of that support was not used for prevention research; as a result, this restriction is unlikely to have affected the findings presented herein. Another potential limitation is that our portfolio analysis excluded basic, preclinical, and clinical research that might eventually lead to prevention research. We cannot know in advance whether such research will ultimately lead to prevention research; therefore, we focused on primary and secondary prevention research in humans, together with related methods research, which is easily identified using the ODP coding methods. Another limitation is that a project was considered prevention research even if only one of its specific aims qualified as prevention research. This limitation would not affect the number of projects but could have inflated the dollar value for prevention research. In addition, this analysis was restricted to research supported by the NIH, and it would be of interest for other funding agencies and foundations to examine their support for prevention research as it is related to the leading risk factors and/or causes of death in the United States.

## Conclusions

Many factors determine how funds for grants and cooperative agreements are allocated to address different health conditions and risk and protective factors across the research spectrum at the NIH. These factors include public health needs, scientific opportunities, the quality of the research applications submitted, and the staffing and infrastructure to support award administration.^1,21^ No level of support for prevention research relative to other types of research has been agreed on, and certainly the need for prevention research will depend on the stage of research for a given area (eg, mechanistic research, development of measures, identification of risk factors, or intervention development). In addition, no scheme for distributing prevention research support to specific exposures, outcomes, or populations or among the various types of research, including randomized clinical trials, observational studies, secondary data analyses, and methods research, has been agreed on. Given the disease and disability burden in the United States associated with the leading risk factors and causes of death and disability, the findings reported herein suggest that the nation may benefit from directing more of the prevention research portfolio supported by NIH grants and cooperative agreements to studies that focus on those risk factors and causes, to studies that address multiple risk factors and causes, and to studies that develop and evaluate preventive interventions to address those risk factors and causes. Doing so will require a multifaceted approach, including a shift in how research proposals are written and funded. Extramural researchers would need to submit proposals that address more than 1 leading risk factor or cause of death and to evaluate interventions to address those risk factors or causes. The NIH institutes and centers would need to prioritize projects that address the leading risk factors and causes of death and disability, projects that address multiple risk factors and causes, and projects that propose trials to evaluate interventions to address those risk factors and causes. This shift in prioritization will not be easy but could lead to major innovations and real progress in disease prevention and health promotion.

## Notes

Supplement. **eMethods.** Protocol for Coding Abstracts Using the ODP Prevention Taxonomy **eTable 1.** Prevention Research Where No Leading Risk Factor or Cause of Death Was Measured as an Exposure or Outcome by Study Exposure or Outcome **eTable 2.** Prevention Research Where Leading Causes of Death Were Measured as an Exposure or as an Outcome **eTable 3.** Prevention Research Where Leading Risk Factors for Death Were Measured as an Exposure or as an Outcome **eFigure 1.** Trends in Prevention Research That Measured a Leading Cause of Death as an Exposure or Outcome **eFigure 2.** Trends in Prevention Research That Measured a Leading Risk Factor for Death as an Exposure or Outcome
