# Supplementary material for: Assessment of Prevention Research Measuring Leading Risk Factors and Causes of Mortality and Disability Supported by the US National Institutes of Health
Source: JAMA Netw Open. 2019 Nov 8;2(11):e1914718. doi: 10.1001/jamanetworkopen.2019.14718 (PMC6902772; doi:10.1001/jamanetworkopen.2019.14718)
Supplement: Supplement. — eMethods. Protocol for Coding Abstracts Using the ODP Prevention Taxonomy eTable 1. Prevention Research Where No Leading Risk Factor or Cause of Death Was Measured as an Exposure or Outcome by Study Exposure or Outcome eTable 2. Prevention Research Where Leading Causes of Death Were Measured as an Exposure or as an Outcome eTable 3. Prevention Research Where Leading Risk Factors for Death Were Measured as an Exposure or as an Outcome eFigure 1. Trends in Prevention Research That Measured a Leading Cause of Death as an Exposure or Outcome eFigure 2. Trends in Prevention Research That Measured a Leading Risk Factor for Death as an Exposure or Outcome [file jamanetwopen-2-e1914718-s001.pdf]

Supplementary Online Content

Vargas AJ, Schully SD, Villani J, Caballero LG, Murray DM. Assessment of prevention research measuring leading risk factors and causes of mortality and disability supported by the US National Institutes of Health. *JAMA Netw Open.* 2019;2(11):e1914718. doi:10.1001/jamanetworkopen.2019.14718

eMethods. Protocol for Coding Abstracts Using the ODP Prevention Taxonomy

eTable 1. Prevention Research Where No Leading Risk Factor or Cause of Death Was Measured as an Exposure or Outcome by Study Exposure or Outcome

eTable 2. Prevention Research Where Leading Causes of Death Were Measured as an Exposure or as an Outcome

eTable 3. Prevention Research Where Leading Risk Factors for Death Were Measured as an Exposure or as an Outcome

eFigure 1. Trends in Prevention Research That Measured a Leading Cause of Death as an Exposure or Outcome

eFigure 2. Trends in Prevention Research That Measured a Leading Risk Factor for Death as an Exposure or Outcome

This supplementary material has been provided by the authors to give readers additional information about their work.

© 2019 Vargas AJ et al. *JAMA Network Open.*

eMethods. Protocol for Coding Abstracts Using the ODP Prevention Taxonomy

Background

The purpose of this protocol is to create a new system to categorize prevention research to allow the NIH Office of Disease Prevention (ODP) to identify and track various types of prevention research in more detail than is possible using current methods. This document provides guidance, including definitions and examples, for categorizing studies based on their abstracts and titles using the accompanying form. It will be used to categorize awards by hand; the data will used to develop a computerized taxonomy coding system.

The lettered items are categories for classification; the numbered items are topics within each category.

General Instructions

- Read the entire abstract before doing any coding, marking passages relevant to each of the categories. Then, go back and read it again in order to code.
- It may be easier to code categories in the order they appear in the abstract, not the order they appear on the form.
- For all categories, please select all the topics that apply to the particular study (except for F. Prevention research category, where you cannot select anything else if you select *6. Other or unclear*)—you will often need to select more than one topic within a category.
- Make coding decisions based on the content of the entire abstract, including the Public Health Relevance section, and on the guidance provided in this document. Do not use other resources (e.g., Google, Wikipedia, etc.).
- Make sure each category on the form has at least one selection.
- As a general rule, code all specific aims for A.2. Exposure and A.3. Outcome. However, these categories may not apply for some specific aims because they may not describe an exposure and outcome being studied. For example, sometimes a first aim refers to developing an intervention, which would not be coded, while the remaining aim(s) test the intervention which was developed, and those aims would typically be coded. Another situation which arises is when aims refer to research capacity building (do not code) or describe what should be coded as E. Study design/purpose.
- If the abstract does not provide enough information about a category, **do not make assumptions** —please select *Other or unclear*.
- If the abstract does not provide enough information to select a topic or there are unresolvable differences within the coding team, select *Other or unclear* for the category. If the abstract has no aims to code and it is not basic research, select *Other or unclear* for A.2. Exposure and A.3. Outcome.
- Basic research includes studies directed toward fundamental knowledge/basic discovery and/or greater understanding of biological structure, biological mechanisms, or behavioral mechanisms. If a study does not identify a measured health outcome (disease, disorder, injury, disability) or risk/protective factor, then it ***may*** be considered basic research. Studies of the pathophysiology of disease ***may*** be considered basic research. Studies that take place in a laboratory paradigm are considered basic research. Pre-clinical studies take place before any testing in humans and can include studies developing a drug, treatment, vaccine, or procedure, as well as studies testing safety or efficacy. Basic research studies and pre-clinical studies are too distal to be considered primary or secondary prevention research. For these studies, please select *Other or unclear* for E. Study design/purpose and *Other or unclear* for F. Prevention research category. You do not need to code the other categories for these abstracts. However, if the abstract also includes another aspect of the study that is primary or secondary prevention research, then do not code the basic research or pre-clinical aspect of the study for any of the categories; instead, code the topics that relate to the aspect that is primary or secondary prevention research.

© 2019 Vargas AJ et al. *JAMA Network Open.*

A. Study Focus

This category includes 3 subcategories, each indicated by a column on the form. For each topic, three columns are provided: A.1. Rationale, A.2. Exposure, and A.3. Outcome. Every column should have at least one selection, so select *Other or unclear* when none of the other topics fits. Certain topics are blacked out because they are not relevant to the column, or because we are not interested in them.

A.1. **Rationale**: This category is used to code the health condition(s) (disease, disorder, injury, disability) or risk/protective factor(s) (that are not blacked out) that the abstract identifies as the motivation for the prevention research. This would be the health condition(s) which the study addresses. The rationale is often stated in the opening few sentences of the study abstract; in other cases, the abstract gives the rationale in the last few sentences. Topics are blacked out if they are not conditions that could serve as the rationale for the study and should be ignored. If a study cites a health condition that is not listed and is not blacked out, select *Other or unclear*. If the abstract does not identify a **health condition** as the rationale, select *Other or unclear*. Do not code unspecified health conditions (e.g. unnamed disability, "other health conditions," "other diseases") in Rationale.

This is the only category on the form that relates to the big picture for the study. Every other category relates to what this specific study will be doing.

For a study of physical activity, the abstract may say that physical activity is important because it is related to heart disease, cancer, and diabetes; in that case, select *Heart disease*, *Cancer*, and *Diabetes* in the rationale column. A study to evaluate a new smoking cessation method may not refer to any specific disease and instead identify only smoking itself as the rationale for the study; in that case, select *Tobacco* as the rationale. Other studies may identify smoking and diseases such as cancer or heart disease together as the rationale – in that case, select *Tobacco* and *Cancer* or *Heart disease* as the rationale. In some cases, mortality (death) is mentioned without identifying the specific cause. For example, the abstract might state how many deaths per year are attributable to a health risk behavior – unhealthy eating, inactivity, tobacco use, alcohol use, etc. Or the abstract might refer to all-cause mortality. In these cases, select *Mortality* as the rationale.

A.2. **Exposure**: This category is used to code the independent variable(s) for the study. There may be more than one exposure that serves as an independent variable for a given study, especially if the study includes several sub-studies. In general, the independent variables are found in the specific aims of the abstract.

For an intervention study, the intervention is the exposure, and the coding should reflect the nature of the intervention. An intervention refers to any kind of exposure designed or intended to have a specific, non-transient effect; examples include surgery to remove cancerous tissue (select *Surgery*), health education programs to encourage smoking cessation (select *Education/counseling*), and medication to treat high blood pressure (select *Medication/device*). This includes interventions delivered under the control of the investigator as well as interventions that are part of a natural experiment. We are looking for the intervention component(s) that differ between the intervention and control group. If both groups get the same background treatment (for example medication or education), then that component of the treatment is not the independent variable. We are only interested in what differs between the groups because that difference is what is being tested. We do not code the control condition under A. Study Focus regardless of the content; code the control condition as appropriate for the other categories.

For an observational study that has no intervention, we are looking for the exposure(s) being measured in the study. For example, groups of people who have different levels of exposure to some possible risk/protective factor for a disease may be compared to see if their health outcomes differ.

This category is applicable to both human and non-human research. If an animal, cell, or tissue is exposed to something as part of the research, that something would be selected as the exposure. For example, animals could be given an experimental medication—in that case, *Medication/device* is the exposure. Or an investigator could monitor the relationship between an animal's diet and the risk of subsequent

© 2019 Vargas AJ et al. *JAMA Network Open.*

disease—in that case, *Diet/nutrition* is the exposure. Or the investigator could expose a tissue to a toxic substance — in that case, *Chemical/toxin* is the exposure.

If more than one exposure is being examined in the study, then select all the exposures. For example, if a study is examining the relationship of blood pressure, physical activity, and obesity to subsequent stroke, select all three (*Blood pressure*, *Physical activity*, and *Obesity*) as the exposures and *Stroke* as the outcome. If a study includes measures of attitude, knowledge, beliefs, or intentions about a certain topic as an exposure, but not a measure of the topic itself, select *Other or unclear* as an exposure. If a measure is described as a perceived exposure or outcome and it is equivalent to a self-report of that exposure or outcome, code the topic as appropriate.

Predictive models of any kind (e.g., statistical, mathematical, computational, simulation) can often be coded in A. Study focus. Predictive models make an inference about the value of an outcome of interest, provided that there are data or information on certain input variables. For example, one or more risk factors can be used to predict a health outcome. Code the predictor variables (inputs) as the exposures and the topic being predicted as the outcome. In some cases, the predictive models will be developed and validated for use by others who have their own input variables; for these studies, select E.2 and F.5 for *Methods research*. Predictive models should not be confused with animal models or theoretical or logic models and frameworks.

Often, investigators are either using or developing new tools to measure disease or disease risk. An abstract may describe the development of a new tool as one of the objectives of a study. In general, do not code a tool itself as exposure or outcome. Rather, code the health condition (disease, disorder, injury, disability) the tool is designed to measure as the outcome. If the way the health outcome is being measured appears in the list, code it as the exposure; if it does not appear, select *Other or unclear*. For example, if cholesterol is used as a measure of heart disease, code *Cholesterol* as the exposure; if stress is measured as an indicator of a mental health condition, code *Stress* as the exposure; if genetics are used to measure musculoskeletal disease, select *Genetics* as the exposure.

The abstract may identify **effect modifiers**, also known as moderators, or describe analyses that will test interactions between the exposure and another variable to see if the effect of that exposure varies according to the level of the other variable. These are all different ways of talking about effect modifiers: variables that modify the effect of the exposure on the outcome. For example, if men respond more than women to the same exposure, then gender is an effect modifier of the relationship between the exposure and the outcome. The analysis of effects in subgroups, or subpopulations, is an example of effect modification, as the purpose of the analysis is to determine whether the effect of the exposure depends on the subgroup or subpopulation. These subgroup analyses may also be referred to as **stratified analyses**, which also allows for the assessment of modifying effects. Analysis of the joint effect of two variables is also an example of effect modification, as its purpose is to determine if the effect of one variable depends on the level of the other. If a variable is being evaluated only as an effect modifier, do not select it as an outcome or as an exposure; however, if a variable is being evaluated both as a main effect and as an effect modifier, code its role as a main effect. For an aim or statement that is exclusively about moderators, do not code it under any category. An exception is gene-environment interactions, where the main effects need to be captured; for these studies, select *Genetics* and the appropriate topic(s) for the environmental exposure as the exposures.

The abstract may identify **confounders**. These are variables that are related to the exposure and to the outcome but not on the causal pathway between them. For example, in a randomized trial to evaluate the effect of smoking cessation on the risk of a first heart attack, there might be more men in the one arm of the study than in the other arm, so that gender is related to exposure just by chance. We know that men are more likely to have a first heart attack than women, so gender is also related to the outcome. As a result, gender is a potential confounder because the uneven distribution of gender across the levels of the exposure could artificially magnify or hide the true effect of the exposure on the outcome. If a variable is being evaluated as a confounder, do not select it as an outcome or as an exposure. For an aim or statement that is exclusively about confounders, do not code it under any category.

© 2019 Vargas AJ et al. *JAMA Network Open.*

The abstract may identify **mediators**. These are variables that are on the causal pathway between the exposure and the outcome. They look just like potential confounders, but we have prior reason to believe that they may be on the causal pathway. For example, if the smoking cessation program works by changing the participant's self-efficacy for quitting, then we would see not only higher levels of quitting but also higher levels of self-efficacy for quitting in the exposed arm. So self-efficacy for quitting would be a potential mediator, because it would be related to the exposure and to the outcome, but it would be on the causal pathway between them. Research on disease or behavioral mechanisms usually involves mediation, as the goal of the research is to identify variables that are on the causal pathway between an exposure and an outcome. If a variable is being evaluated as a mediator, do not select it as an outcome or as an exposure. For an aim or statement that is exclusively about mediators, do not code it under any category.

A.3. Outcome: This category is used to code <u>the dependent variable in the study</u>, which may differ from the rationale and the exposure. In general, the dependent variables are found in the specific aims of the abstract.

Most of the topics under A. Study Focus could be exposures or outcomes, depending on the purpose of the study. For example, for a study examining the relationship between the level of physical activity and mortality, *Physical activity* is the exposure and *Mortality* is the outcome. For a study examining the effects of a counseling intervention on physical activity level, *Education/counseling* is the exposure and *Physical activity* is the outcome. The abstract may say "physical activity is important for heart disease, cancer, and diabetes," but if the study is not measuring any of those conditions, then do not select them as outcomes; instead, select them under rationale).

Sometimes a study starts by identifying people with the health condition of interest and looks to see what they have been exposed to. These are case-control studies and the health condition, though appearing first in this abstract, is the outcome.

If an abstract says they will measure feasibility, acceptability, or a similar term, code those as *Other or unclear* outcomes. An exception is if a study is measuring side effects, safety, or toxicities of a medication/device – these should be coded as *Medication/device* for the outcome. For these studies, also select *Pilot/feasibility/proof-of-concept/safety* under E. Study design/purpose. If a study includes measures of attitude, knowledge, beliefs, or intentions about a certain topic as an outcome, but not a measure of the topic itself, select *Other or unclear* as an outcome. For example, if a study includes a measure of quitting self-efficacy as an outcome, select *Other or unclear* to represent that outcome instead of *Tobacco*; if the study also includes a measure of tobacco use as an outcome, select *Tobacco* as well as *Other or unclear*.

The abstract may identify **effect modifiers**, also known as moderators, or describe analyses that will test interactions between the exposure and another variable to see if the effect of that exposure varies according to the level of the other variable. These are all different ways of talking about effect modifiers: variables that modify the effect of the exposure on the outcome. For example, if men respond more than women to the same exposure, then gender is an effect modifier of the relationship between the exposure and the outcome. The analysis of effects in subgroups or subpopulations is an example of effect modification, as the purpose of the analysis is to determine whether the effect of the exposure depends on the subgroup or subpopulation. These subgroup analyses may also be referred to as **stratified analyses**, which also allows for the assessment of modifying effects. Analysis of the joint effect of two variables is also an example of effect modification, as its purpose is to determine if the effect of one variable depends on the level of the other. If a variable is being evaluated only as an effect modifier, do not select it as an outcome or as an exposure; however, if a variable is being evaluated both as a main effect and as an effect modifier, code its role as a main effect. For an aim or statement that is exclusively about moderators or, do not code it under any category. An exception is gene-environment interactions, where the main effects need to be captured; for these studies, select *Genetics* and the appropriate topic(s) for the environmental exposure as the exposures.

The abstract may identify **confounders**. These are variables that are related to the exposure and to the outcome but not on the causal pathway between them. For example, in a randomized trial to evaluate the

effect of smoking cessation on the risk of a first heart attack, there might be more men in the one arm of the study than in the other arm, so that gender is related to exposure just by chance. We know that men are more likely to have a first heart attack than women, so gender is also related to the outcome. As a result, gender is a potential confounder because the uneven distribution of gender across the levels of the exposure could artificially magnify or hide the true effect of the exposure on the outcome. If a variable is being evaluated as a confounder, do not select it as an outcome or as an exposure. For an aim or statement that is exclusively about confounders, do not code it under any category.

The abstract may identify **mediators**. These are variables that are on the causal pathway between the exposure and the outcome. They look just like potential confounders, but we have prior reason to believe that they may be on the causal pathway. For example, if the smoking cessation program works by changing the participant's self-efficacy for quitting, then we would see not only higher levels of quitting but also higher levels of self-efficacy for quitting in the exposed arm. So self-efficacy for quitting would be a potential mediator, because it would be related to the exposure and to the outcome, but it would be on the causal pathway between them. Research on disease or behavioral mechanisms usually involves mediation, as the goal of the research is to identify variables that are on the causal pathway between an exposure and an outcome. If a variable is being evaluated as a mediator, do not select it as an outcome or as an exposure. For an aim or statement that is exclusively about mediators, do not code it under any category.

Topics

Below are examples and definitions for each of the topics for A. Study Focus. A number of health conditions are available as possible rationales for the prevention research. These include the ten leading causes of death based on 2010 CDC data and several of the actual causes of death identified by Mokdad et al. (2004). All topics except *Maternal/paternal/child health* and *Mortality* are available as possible exposures, as death cannot be a precursor to another health condition. Additionally, *Maternal/paternal/child health* cannot be an outcome. Often abstracts will refer generally to the disease burden, or to morbidity and mortality, as part of the rationale for the study. If the abstract names mortality or death in this way, then select *Mortality* under A.1. Rationale. But do not select other topics in lieu of "morbidity." All topics are available as possible outcomes.

An abstract may identify disease risk as an exposure or outcome. Select the topic that represents that disease as the exposure or outcome only when risk is based on a measure of the health condition itself (i.e., incidence or prevalence of the condition). Otherwise, select *Other or unclear* for disease risk. If a study is examining prevalence or incidence of a health condition (disease, disorder, injury, disability) or risk/protective factors as one of its aims, select *Other or unclear* as the exposure and the health condition, as the outcome. If a study describes symptoms that are not diagnostic of a disease or condition (e.g. depressive symptoms, psychiatric symptoms, diabetes symptoms, coughing as a symptom), select *Other or unclear* as appropriate for exposure or outcome. An abstract may study self-management of a disease (e.g. diabetes self-management, severe mental illness self-management); in these cases, select the health condition or disease as appropriate for exposure or outcome. If a study is using an established and validated proxy measure as an exposure or outcome, select the topic being measured and the topic for which the measure is a proxy for exposure or outcome as appropriate.

In methodological studies, when an investigator is using an established and validated screening tool, the screening tool is classified as the disease for which it is intended to screen. For example, select *Neurological disease* (not *Alzheimer's disease*) for an established and validated screening tool for autism spectrum disorders or *Suicide* for an established and validated screening tool that assesses suicidal ideation. If a methodological study is developing, evaluating, and/or validating a new screening tool/measure or applying an established screening tool in a new population, select *Other or unclear* for the exposure or outcome as appropriate.

1. *Alcohol*
   Alcohol use/misuse is the 3rd actual cause of death and is a diagnosable condition, so it can be a rationale. Alcohol use/misuse can cause liver disease, motor vehicle crashes, and other conditions that cause morbidity (e.g., injuries) or are fatal. This topic includes measures of alcohol use/misuse such as age of onset, number of drinks per day or week, and blood alcohol level. It could be an exposure if, for example, a study is interested in whether alcohol use is related to risk of stomach cancer or some other health

© 2019 Vargas AJ et al. *JAMA Network Open.*

condition. It could be an outcome if a study is interested, for example, in whether an intervention program reduces binge drinking or some other measure of alcohol use by college students. For studies that examine alcohol dependence disorder as an exposure or outcome, select *Alcohol* and do not select *Mental Health*.

2. *Alzheimer's disease*
   Alzheimer's disease is the 6th leading cause of death and can be a rationale. Because of its prominence, we are interested in it separately from other neurological conditions. It could be an exposure if we are interested, for example, in whether Alzheimer's disease increases the risk of another health condition. It could be an outcome if we are interested, for example, in whether another exposure increases the incidence of Alzheimer's disease.

3. *[Removed]*

4. *Blood disorder*
   This includes blood disorders like anemia or hemophilia, sickle cell anemia. For blood cancers like leukemia, select *Cancer* instead. Blood pressure is not a blood disorder. Blood disorders are health conditions, so they can be the rationale. Blood disorders could be an exposure if we are interested, for example, in whether those with sickle cell anemia are more likely to develop a particular cancer. Blood disorders could be an outcome if we are interested, for example, in whether those with a particular genetic variation are more likely to have sickle cell anemia. For studies that examine sepsis as an exposure or outcome, select *Infectious disease* and do not select *Blood disorder*.

5. *Blood pressure (BP)*
   Elevated blood pressure (hypertension) is a risk factor for heart disease, stroke, and kidney disease. We do not consider it separately to be a health condition, so it cannot be a rationale. It can be an exposure if the study is looking at, for example, how BP is related to subsequent disease, or an outcome if a study is looking at, for example, what factors or interventions influence BP or hypertension.

6. *Cancer*
   Cancer is the 2nd leading cause of death and can be a rationale. This topic includes screening or diagnosis of any form of cancer (including blood cancers like leukemia), malignancies/malignant tumors or neoplasms. This topic does not include precancerous conditions/lesions (e.g., ductal carcinoma in situ (DCIS) or CIN1-3 cervical dysplasia); select *Other or unclear* instead. For lung cancer, for example, select *Cancer*, not *Lung disease*. It would be an exposure if the researchers are interested, for example, in whether diagnosis with a particular cancer increases the risk of another health condition. It would be an outcome if we are interested, for example, in whether exposure to a toxin increases the incidence of cancer. For studies that examine family history of cancer as an exposure, select *Other or unclear* and not *Cancer*.

7. *Chemical/toxin*
   Toxins are the 5th actual cause of death. This topic includes toxicants, air pollution, water pollution, environmental radiation (e.g., radon), metals, endocrine disruptors, dietary toxins, and others. It is not a health condition, so it cannot be a rationale. It includes measures of exposure to chemical/toxins, such as mercury levels in the blood. This topic does not include UV radiation (selection *Other or unclear* instead). It can be an exposure, for example, in studies examining the association between exposure to a particular chemical and risk of developing a health condition. It can be an outcome, for example, in studies examining the relationship between dietary intake and levels of a certain toxin in the blood. If a study is examining a specific toxin or chemical, select *Chemical/toxin* as an exposure or outcome and not the source of a toxin. If the study is examining the source of the toxin and not the individual components, select the source(s) of the toxin (e.g., *Diet/nutrition* for dietary intake, *Other or unclear* for environment, or *Tobacco* for tobacco smoke) as an exposure or outcome. If a study is examining toxicities of medications, select *Medication/device*. If the source is an important motivation for the study then it may be appropriate to select it as the rationale if it is a health condition (e.g., *Tobacco* or *Substance abuse*). If, for example, a study examines allergies to latex, then select *Chemical/toxin* as the exposure and *Other or unclear* as the outcome.

8. *Cholesterol*

© 2019 Vargas AJ et al. *JAMA Network Open.*

This topic is about blood cholesterol, not dietary cholesterol. Elevated blood cholesterol is a risk factor for (not a diagnostic measure of) heart disease, stroke, and other vascular diseases. This topic includes all forms and measures of cholesterol found in the blood, including LDL-cholesterol, HDL-cholesterol, lipoproteins, triglycerides, LDL size, blood lipid panels, etc. We do not consider it separately to be a health condition, so it cannot be a rationale. It can be an exposure if we are interested, for example, in whether those with elevated cholesterol are at risk for a health condition. It can be an outcome if we are interested, for example, in whether those who are very active physically have lower cholesterol.

9. *Diabetes*
Diabetes is the 7th leading cause of death and so it can be a rationale. This topic includes both type 1 or type 2 diabetes mellitus and gestational diabetes mellitus. It also includes self-care or self-management of diabetes (e.g., glycemic control), as well as diagnostic measures of diabetes, such as fasting blood glucose levels, impaired glucose tolerance, or glycated hemoglobin (Hb A1c) levels. This topic does not include metabolic/cardiometabolic disorders or pre-diabetic states (e.g., glucose intolerance, insulin resistance, pre-diabetes); select *Other or unclear* for these conditions. It can be an exposure if the study is asking, for example, whether diabetes increases the risk of another disease, such as a cardiovascular disease. It can be an outcome if the study is asking, for example, whether an exposure like a lifestyle intervention reduces the rate of diabetes.

10. *Diet/nutrition*
Diet is the 2nd actual cause of death. It is not a health condition, so it cannot be a rationale. This topic includes dietary composition (e.g., macronutrients, micronutrients, calorie/energy intake), diet quality scores and dietary patterns, dietary supplements, dietary behaviors (excluding anorexia and bulimia, which are disorders and included in *Mental Health*), food or beverage purchases, and compliance to a specific diet or dietary supplement, etc. Dietary supplements include vitamins, minerals, herbs or other botanicals, amino acids, and other dietary ingredients or their constituents, and exclude tobacco products. Dietary supplements are intended for a healthy consumer population, whereas medications are specially formulated for a diseased patient population and make health claims. Studies examining omega-3 fatty acids or caffeine metabolites are included in this topic. This does not include malnutrition (select *Other or unclear*) or ingested toxins (select *Chemicals/toxin*). We are interested in diet/nutrition as it relates to disease; therefore the disease(s) of interest is the rationale. It can be an exposure, for example, in a study examining whether a specified diet will reduce risk of cardiovascular disease. It can be an outcome, for example, in a study that investigates whether a certain intervention will lead to people choosing to eat healthier food. It may be appropriate to select *Diet/nutrition* for the exposure, or for the outcome, or both. If, for example, a study examines allergies to nuts, then it may be appropriate to select *Diet/nutrition* as the exposure and *Other or unclear* as the outcome. If, for example, a study is examining the effect of lactose intolerance on gastrointestinal diseases, then it may be appropriate to select *Diet/nutrition* as the exposure and *Gastrointestinal disease* as the outcome.
If a study includes measures of attitude, knowledge, beliefs, or intentions about diet/nutrition, select *Other or unclear*.

11. *Education/counseling*
This topic includes conveying knowledge, advice, or information, providing or studying social support, or providing behavioral counseling or skills training, regardless of the delivery mode (could be in person, over the phone (e.g., quitlines), by internet, by smart phone, in groups, individually, etc.—all of these delivery modes are included in this topic). Other indications that a study is examining an education/counseling intervention include terms such as training, feedback, or therapy (e.g., behavior modification, psychotherapy, psychoanalysis, hypnosis, motivational interviewing, cognitive or cognitive- behavioral therapy, mindfulness, self-help materials, peer-led interventions, or school-based curriculum). If a study is examining contingency management or providing rewards/incentives, select *Other or unclear*, unless another part of the intervention leads you to select *Education/counseling*. *Education/counseling* cannot be a rationale because it is not a health condition. Rather, *Education/counseling* can be an exposure for an observational study, or a type of intervention for an intervention study. It can be an outcome if the study is looking at how to improve an educational system (e.g. changing a curriculum), what factors are associated with the delivery of education, or what factors impact academic performance. If a study is examining attitudes, intentions, or beliefs, select *Other or unclear* as appropriate. If a study is measuring retention of

© 2019 Vargas AJ et al. *JAMA Network Open.*

knowledge resulting from an intervention, select *Education/counseling* as the outcome. Screening, Brief Intervention, and Referral to Treatment (SBIRT) interventions, as the name suggests, include a screening component, a brief, education/counseling intervention, and a referral to treatment component. For studies examining the effect of SBIRT interventions, select *Education/counseling* as the exposure. In addition, if a validated screener is being used as part of the SBIRT intervention, select the health condition that is being screened as the exposure (e.g., *Alcohol*, *Substance abuse*, or *Tobacco*).

12. *Firearms*
Firearms are the 7th actual cause of death. Firearms are not a health condition, so cannot be a rationale. This topic includes carrying, making threats with, or using firearms. Firearms can be an exposure that causes mortality or injuries, or an outcome if a study is looking at how to reduce use of firearms. If a study includes measures of attitude, knowledge, beliefs, or intentions about firearms as outcomes, select *Other or unclear* to represent those outcomes. If a study about firearms also has specific aims related to violence without firearms, select *Violence* for the exposure or outcome as appropriate.

13. *Gastrointestinal disease (GI)*
GI diseases include conditions of or related to the GI system (i.e., esophagus, stomach, intestines, liver, pancreas, gall bladder, spleen), such as duodenal ulcers, GI bleeding, Barrett's esophagus; Crohn's disease, inflammatory bowel disease, etc. For GI cancers, select *Cancer* instead. For virus- or bacteria-induced GI disorders (e.g., hepatitis, ulcers caused by *H. pylori*), select *Infectious disease* instead. Diarrhea and ulcers of unclear/non-specified etiology are included in this topic. It is a health condition, so it can be a rationale. GI disease can be an exposure if, for example, a study is interested in whether people with stomach ulcers are more likely to develop anemia. GI disease can be an outcome if a study is interested, for example, in whether people who take a particular dietary supplement are more likely to develop duodenal ulcers. If, for example, a study is examining lactose intolerance, then it may be appropriate to select *Diet/nutrition* as the exposure and *Gastrointestinal disease* as the outcome.

14. *Genetics*
Relevant study types include genome-wide association studies (GWAS), genomics, and epigenetics (i.e., the study of processes that regulate how and when certain genes are turned on and off). Genetics are not a health condition, so it cannot be a rationale. Genetics can be an exposure, for example, in a study that evaluates a genetic variant as a possible risk factor for developing a disease (e.g., BRCA1/BRCA2 for breast cancer or ApoE for Alzheimer's disease). For GWAS, which seek to identify genetic variants that are associated with health outcomes, select *Genetics* as the exposure and the disease or health condition as the outcome. Genetics could be an outcome, for example, in a study examining the effects of an intervention on indicators of epigenetic processes such as DNA methylation. *Genetics* should not be selected just because the study is using genetic material to identify the entities studied. For example, do not select *Genetics* for a study about the microbiome where genetic material is being used only to identify the individual microbes in the microbiome (select *Microbiome* for the rationale, exposure, or outcome) as appropriate. Select *Other or unclear* for studies involving congenital diseases that affect multiple organ systems (e.g., Down syndrome or cystic fibrosis) as the rationale, exposure, or outcome as appropriate, unless the study focus is on screening for or incidence of congenital diseases; in those cases, select *Maternal/paternal/child health* for rationale. For studies examining gene-environment interactions, select *Genetics* and *Other or unclear* for the environmental component, unless the environmental component is specified in the abstract (e.g. *Tobacco, Chemical/toxin*).

Terms often used in genetic research include gene expression (i.e., the process by which the information encoded in a gene is used to direct the assembly of a protein molecule); gene regulation (i.e., the process of turning genes on and off); gene modulation (i.e. the practice of altering the expression of a gene); and single nucleotide polymorphisms (SNPs) (i.e., the most common type of genetic variation among people). Other common terms associated with genetic research include alleles, chromatin, cloning, codon, DNA, exome, genome, heterozygous, histone modification, homozygous, imprinting, inherited, messenger RNA (mRNA), mutation, oncogene, polymorphism, promoter, RNA, recessive, and telomere. Terms that should not be coded in this topic (select *Other or unclear* instead) include amino acid, antibody, enzyme, histone, metabolome/metabolomics, phenotype, protein, proteome/proteomics, transcription factor, and microbiome Note: proteins are the product of gene translation and should not be coded under *Genetics*.

© 2019 Vargas AJ et al. *JAMA Network Open.*

15. *Healthcare delivery*

This topic includes access to healthcare services, utilization of healthcare services, healthcare financing and costs (e.g., health insurance payments and out of pocket expenses such as deductibles and co-payments), organization of care, and quality of care. It also includes studies of clinical practice or aspects of the healthcare system such as patient-centered medical homes, decision-support systems, and healthcare teams. It may include studies of electronic health records if they are used to measure healthcare utilization. *Healthcare delivery* cannot be a rationale; the disease/condition for which the care is delivered is the rationale. This topic does not include studies of medical devices or pharmaceuticals (select *Medication/device*), or cost-effectiveness of interventions (select *Other or unclear*).

It can be an exposure, for example, if the study is asking whether providing a certain healthcare service is associated with improved utilization and/or better patient outcomes. For example, in a study examining whether physicians are using a risk calculator to determine patients' risk for a stroke is associated with fewer strokes, select *Healthcare delivery* as the exposure. Select this topic, for example, as an exposure for a study testing the use of a clinical decision support tool to improve screening for diabetes. For a study to assess whether using a team approach to reduce cardiovascular risk in patients with diabetes works better than not using a team approach, select *Healthcare delivery* for the exposure. For a study to ascertain whether changes in health insurance or patient cost sharing impact patient outcomes, select this topic as the exposure. This topic does not include testing education or counseling interventions delivered by healthcare personnel (select *Education/counseling*), except where the mode of delivery is being tested (e.g. all study participants receive the education/counseling intervention, but it is delivered in one arm by trained lay staff and in another arm by nurses or physicians); for these studies, select only *Healthcare delivery* for the exposure.

It can be an outcome, for example, if a study is testing an intervention to improve the availability, utilization, cost, or quality of healthcare services. Select this topic for a study measuring utilization of healthcare services (e.g., medical procedures, screenings, vaccinations) delivered by a clinician (e.g., physicians, nurses, or other registered/licensed/trained healthcare professionals). For example, screenings that are always performed by a clinician such as mammograms, sigmoidoscopies, and colonoscopies are included in this topic. Tests that do not involve or are not ordered by a clinician are not considered *Healthcare delivery* (e.g., select *Genetics* for at-home genetic tests or select *Other or unclear* for home pregnancy tests).

If a study refers to a specific type of service utilization, then select both *Healthcare delivery* and the topic associated with the type of service (e.g., *Kidney disease* for dialysis service use, *Infectious disease* for HIV testing, etc.). Studies of healthcare service utilization can be in traditional health care settings (e.g., doctors' offices, hospitals, clinics) as well as in nontraditional health care settings (e.g., health fairs, churches, pharmacies). For a study evaluating the effectiveness of an intervention in promoting increased utilization of mental health services, select both *Healthcare delivery* and *Mental health* for the outcomes. In a study of the impact of an intervention on patient out-of-pocket costs, select *Healthcare delivery* for the outcome. Select this topic, for example, as an outcome for a study testing whether providing flu shots reduces hospitalizations. For a study to assess whether an intervention increases referrals to care, select *Healthcare delivery* as the outcome. This topic does not include routine clinical measurements such as height, weight, and blood pressure (select *Other or unclear* for these outcomes).

16. *Heart disease*

Heart disease is the leading cause of death and can be a rationale. This topic includes cardiac conditions such as heart attacks (myocardial infarction), heart failure, arrhythmias, atherosclerosis of the heart, heart valve conditions, angina, cardiac or cardiovascular events, and structural abnormalities/defects of the heart. For studies about heart disease mortality or sudden cardiac death, it may be appropriate to select *Heart disease* as the rationale and *Mortality* as the outcome. Heart disease can be an exposure, for example, in a study to find out if patients with a prior heart attack are more likely to develop a particular form of dementia. Heart disease is often an outcome, for example, in a study examining the relationship between dietary factors and heart attacks. If a study cites cardiovascular disease (CVD) or peripheral artery disease

(PAD) as a rationale, exposure, or outcome, select both *Heart disease* and *Stroke*; but select only *Heart disease* if a study cites coronary heart disease (CHD) as a rationale, exposure, or outcome. If a study cites cardiometabolic disease as a rationale, exposure, or outcome, select *Other or unclear*. This topic does not include pre-clinical or subclinical cardiovascular disease (e.g., cardiac remodeling, which includes changes in size, shape, or function of the heart in response to disease); select *Other or unclear*.

17. *HRQOL (Health-related quality of life)*
    This topic refers to quality of life, usually associated with aging or a specific medical condition (e.g., HRQOL specific to breast cancer patients). HRQOL is a multi-dimensional concept that includes domains related to functional status such as physical, mental/cognitive, emotional, social functioning, and disability. This topic does not include cognitive decline (select *Other or unclear*). It goes beyond direct measures of population health, life expectancy, and causes of death, and focuses on the impact health status has on quality of life. Key elements used to measure HRQOL include activities of daily living (ADL), instrumental activities of daily living (IADL), quality-adjusted life years (QALYs), and disability-adjusted life years (DALYs). It is not a health condition, so it cannot be a rationale. Sometimes a study measures HRQOL as one of its outcomes, rarely as an exposure.

    A distinct but related concept is well-being, which assesses the positive aspects of a person's life, such as positive emotions and life satisfaction. Well-being is a relative state where one maximizes his or her physical, mental, and social functioning in the context of supportive environments to live a full, satisfying, and productive life. Select *Other or unclear* for studies that examine well-being.

    Another similar concept is cognitive functioning or cognitive decline in the context of assessing aging or mental health. These measures are not diagnostic of a mental health condition, so they are not coded as *Mental health*; instead, they are coded as *Other or unclear*.

18. *Infectious disease*
    This topic includes any infectious disease or agent, whether viral, bacterial, or parasitic. Examples include HIV/AIDS, other sexually transmitted infections, tuberculosis, hepatitis, measles, human papillomavirus (HPV), schistosomiasis, otitis, periodontitis, tropical infectious diseases, and diarrheal disease caused by an infectious disease agent. If the study involves pneumonia/influenza, select that topic instead. This topic also includes testing for infectious diseases (e.g., HIV testing) and measures of infectious disease (e.g. viremia, antibodies, or viral blood count) indicating natural infection or immunity, not vaccine-induced immunity. For studies using measures of infectious disease that indicate vaccine-induced immunity, select *Vaccine* to represent these measures. Select this topic if a study is examining a mutation of the infectious disease agent that leads to drug-resistance or vaccine escape.

    Infectious disease is a health condition, so it can be a rationale, even if no specific infectious disease is named. It can also be an exposure, for example, in studies of whether persons with a particular virus are more likely to develop congestive heart disease. Antibodies are typically a marker for being infected with an infectious disease, so if a study investigates antibodies in this context, select *Infectious disease*. It can be an outcome, for example, in a study testing a possible vaccine against malaria. If the study is examining a vaccine, candidate vaccine, or vaccine components to protect against an infectious disease, it may be appropriate to select *Vaccine* as the exposure and *Infectious disease* as the outcome and/or as the rationale. For studies that examine sepsis as an exposure or outcome, select *Infectious disease* and do not select *Blood disorder*. Studies of immune response are not necessarily coded as *Infectious disease* unless the overall goal of the study focuses on one or more specific infectious diseases. For studies of allergies or immunocompetence as an exposure or outcome, select *Other or unclear*. If a study is examining the impact of antiretroviral therapy (ART) in the prevention of HIV transmission, it may be appropriate to select *Medication/device* as the exposure and *Infectious disease* as the outcome.

19. *Kidney disease*
    Kidney disease is the 8th leading cause of death and can be a rationale. It includes conditions of the urinary system such as chronic kidney disease, nephritis, renal failure, urinary tract infections, and kidney stones. It may also include studies of bladder function, kidney function, and dialysis. It can be an exposure, for example, in a study looking at the risk for hepatitis C infection among persons with and without a history of

© 2019 Vargas AJ et al. *JAMA Network Open.*

urinary tract infections. It can be an outcome, for example, in studies looking at the relationship between a dietary supplement and nephritis. For studies measuring utilization of dialysis services, select *Kidney disease* and *Healthcare delivery*. For cancers involving the kidney, select *Cancer* instead.

20. *Lung disease*
Chronic lung disease is the 3rd leading cause of death and can be a rationale. This includes emphysema, chronic obstructive lung disease (COLD), chronic obstructive pulmonary disease (COPD), chronic lower respiratory disease, pulmonary fibrosis, asbestosis, and asthma. It also includes measures of lung disease such as forced expiratory volume (FEV_1) as measured by spirometry or exhaled nitric oxide (eNO) for asthma. *Lung disease* does not include pneumonia/influenza, infectious disease (e.g., tuberculosis, bronchitis, or pertussis), or cancer; select the appropriate topic. It can be an exposure, for example, in a study looking at whether having asthma affects physical fitness or physical activity. It can be an outcome, for example, in studies looking at whether e-cigarette use is associated with asthma. For cancers in the lung, select *Cancer* instead.

21. *Maternal/paternal/child health*
Although this topic is not a specific health condition, it can be a rationale. If a study uses exposures or outcomes that are included in this topic, select this topic as rationale. This topic includes studies of normal and abnormal growth, development, and adjustment during or after pregnancy, addressing the health of the mother, father, or the fetus/infant/toddler/preschool-aged child (i.e., age 5 and under). It includes developmental studies of the infant/toddler/preschool-aged child, but excludes studies of health conditions that happen to occur during childhood (e.g., childhood obesity, childhood cancer, childhood malaria, childhood asthma). For studies evaluating developmental delays due to autism during childhood, select *Maternal/paternal/child/health* and *Neurological disease* as rationale. The topic also includes studies of reproductive health of men and women including fertility, fecundity, unintended pregnancies, in utero influences (e.g. maternal smoking, maternal stress, maternal diet) on the fetus/infant/toddler/preschool-aged child (i.e., age 5 and under). It also includes studies of preconception (i.e., those designed to improve pregnancy and birth outcomes) and post-partum (i.e., up to one year following birth). This topic includes screening for and incidence of birth defects (e.g., neural tube defects, Down syndrome, cerebral palsy, cystic fibrosis, fetal alcohol syndrome, etc.) and inborn errors of metabolism, also known as congenital metabolic disorders (e.g., phenylketonuria, or PKU). If a study is about fetal alcohol syndrome, select both *Alcohol* and *Maternal/paternal/child/health* as rationale. It also includes parenting (e.g., parental functioning, parent-child interaction) for children through preschool, and child-rearing practices such as breastfeeding and preventing mother-to-child transmission (PMTCT) of infectious disease. This topic includes studies of infants with HIV if transmission occurred in utero, during birth, or post-partum through breastfeeding. This topic excludes parenting decisions related to medical care (e.g., infant vaccination, tonsillectomy). It also includes child maltreatment (which includes neglect and abuse); for studies on child maltreatment specifically involving physical abuse, select *Violence* as the exposure or outcome as appropriate, in addition to *Maternal/paternal/child health* as rationale.

If a study uses contraceptive methods that are medications or devices, select *Medication/device* as the exposure or outcome and *Maternal/paternal/child health* as the rationale, if appropriate. For studies that examine fetal death, miscarriage, abortion, and pregnancy termination, select *Maternal/paternal/child health* for rationale and *Other or unclear* for outcome and not *Mortality* since these data are compiled separately by the CDC National Center for Health Statistics. Select *Maternal/paternal/child health* and *Mortality* for rationale for studies of infant death which occurs before the first birthday (e.g., Sudden infant death syndrome, also known as SIDS).

22. *Medication/device*
This topic includes any medication, or strategy of using multiple medications, as well as medical devices such as defibrillators, artificial valves, screening devices such as a colonoscope, etc. and their side effects or toxicities. It includes medical use of marijuana and nicotine when delivered as a medical product (e.g., nicotine replacement products such as nicotine patch, nicotine inhaler, nicotine lozenge, nicotine nasal spray, or nicotine gum). Note: other tobacco products, such as e-cigarettes, are not medical products and cannot be marketed as such and so do not qualify as medications or medical devices; in these cases, select *Tobacco*. This topic does not include dietary supplements (select *Diet/nutrition*), radiation treatment (if

© 2019 Vargas AJ et al. *JAMA Network Open.*

radiosurgery, select *Surgery*, otherwise select *Other or unclear*), or *Vaccines*. If a study is examining drug-resistance or uses a similar term, select *Infectious disease,* or *Pneumonia/influenza* if it is examining that disease as the rationale, exposure, or outcome as appropriate, because the drug-resistance is caused by changes in the infectious agent.

Medications or devices are not health conditions and so cannot be a rationale. This topic can be an exposure, for example, if a study is evaluating the efficacy of a new medication or medical device in preventing a health condition. Medications are specially formulated for a diseased patient population and make health claims. If a study is examining the effectiveness of a new type of condom, select *Medication/device* as the exposure. This topic can be an outcome, for example, if a study is looking at what exposures or interventions influence the delivery of medications, the use of a medical device in clinical practice, or the sensitivity, pharmacokinetics, or pharmacodynamics of a medication or drug. Another example of *Medication/device* as an outcome is a study that examines measures of medication or device compliance. If a study uses contraception methods that are medications or devices (e.g., IUD, birth control pill/patch, Depo-Provera), select *Medication/device* as the exposure or outcome and *Maternal/paternal/child health* as the rationale, if appropriate. If a study is examining the impact of antiretroviral therapy (ART) in the prevention of HIV transmission, it may be appropriate to select *Medication/device* as the exposure and *Infectious disease* as the outcome. Studies evaluating the side effects or toxicities of a medication/device are evaluating the safety of the medication/device; select *Medication/device* for the outcome and *Pilot/feasibility/proof-of-concept/safety* under E. Study design/purpose for these studies. For example, if a study is investigating allergies to penicillin, then it may be appropriate to select *Medication/device* as the exposure and outcome. If an abstract says they will measure feasibility, acceptability, or a similar term, code those as *Other or unclear* outcomes.

23. *Mental health*
    This topic includes mental health disorders such as mood disorders (e.g., major depressive disorder, anxiety disorders), post-traumatic stress disorder (PTSD), attention deficit disorder, eating disorders (e.g., anorexia, bulimia), conduct disorder, schizophrenia, etc. Select this topic if the investigator uses the term “mental health,” “mental illness,” “depression,” ”psychiatric or psychological disorder,” “psychopathology,” or similar terms but not “psychological health” or “depressive symptoms.” It includes self-care or self-management of psychiatric or mental health disorders. It does not include mental states that are not disorders, such as moods (select *Other or unclear*), or neurological disorders and dementia (select *Neurological disease*), or Alzheimer’s disease (select *Alzheimer’s disease*). It is a health condition, so it can be a rationale. It can be an exposure, for example, in studies of whether people with a particular mental illness are more likely to abuse drugs or become HIV-infected. It can be an outcome, for example, in a study examining environmental or genetic exposures to the development of a certain mental illness. *Mental health* does not include cognitive decline (select *Other or unclear*) or cognitive functioning (may be appropriate to select *HRQOL (Health-related quality of life)*). For studies that examine addictive behavior disorders as an exposure or outcome, select *Alcohol, Substance abuse,* or *Tobacco* as appropriate and do not select *Mental Health*. Note: *Suicide* is a separate topic of its own and therefore is not included as part of *Mental Health*.

24. *Microbiome*
    These are communities of microbes (e.g., bacteria, fungi, archaea, viruses) found at many sites on the human body. These microbes often exist in a symbiotic relationship with their human hosts. This topic includes analysis of the role of these microbes in human health and disease. Therefore, the microbiome would be considered an exposure for studies that examine the relationship between microbiomes and disease. For example, if a study is examining the effect of the microbiome on colorectal cancer, select *Microbiome* as the exposure and *Cancer* as the outcome. This topic can be an outcome if a study is examining the changes of the microbiome due to a health condition, environmental, or other exposure. For example, if a study is examining the effect of physical activity on changes of the gut microbiome, select *Physical activity* as the exposure and *Microbiome* as the outcome. This topic excludes the study of pathogenic or infectious microbes; for these studies, select *Infectious disease.* Studies that are only examining the content of the microbiome (i.e., the individual microbes within the microbiome) with no relation to a health outcome or risk factor are considered basic research.

© 2019 Vargas AJ et al. *JAMA Network Open.*

25. *Mortality*
Mortality and death are synonymous. This topic includes the term "survival" but not necessarily "survivor" or "survivorship." It also includes the terms "longevity" and "lifespan" if used in the context of mortality. It does not include studies that use survival analysis to analyze data unless death is a measured outcome. Measures that use death in their calculation, such as quality-adjusted life years (QALYs) and disability-adjusted life years (DALYs), are included in this topic. *Mortality* can be a rationale; for example an abstract or Public Health Relevance statement may include a statistic about deaths per year. *Mortality* cannot be an exposure because mortality cannot be a precursor to another health condition. *Mortality* can be an outcome, whether due to a specific condition or all-cause mortality. For studies of disease-specific mortality as an outcome, select both the disease and *Mortality* as outcomes. Note: *Suicide* is a separate topic of its own and therefore is not included as part of *Mortality*. For studies that examine fetal death, miscarriage, abortion, or pregnancy termination, select *Maternal/paternal/child health* and not *Mortality* for rationale since these data are compiled separately by the CDC National Center for Health Statistics.

26. *Motor vehicle crash (MVC)*
MVCs are the 6th actual cause of death. This topic includes all types of motor vehicle crashes and all types of motor vehicles: cars, buses, airplanes, etc. It is not a health condition, so it cannot be a rationale. It can be an exposure, for example, in a study of unintentional injuries resulting from motor vehicle crashes. It can be an outcome, for example, in studies examining the effect of a change in policy governing drinking ages and nighttime single vehicle crashes.

27. *Musculoskeletal disease*
This topic includes conditions of the muscles or skeleton (including teeth). Examples include osteoarthritis, osteoporosis, rheumatoid arthritis, other joint problems, myositis, myopathy (though cardiac myopathy is included in *Heart disease*), and dental caries. It is a health condition, so it can be a rationale. It can be an exposure, for example, in a study of whether history of rheumatoid arthritis increases the risk of unintentional injuries. It can be an outcome, for example, in a study to identify genetic risk factors for osteoarthritis. *Musculoskeletal disease* does not include fractures; for these studies, select *Unintentional injuries*.

28. *Neurological disease (not Alzheimer's)*
This topic includes conditions of the nervous system and neurological disorders, e.g., Parkinson's, neuropathies, autism, dementia, epilepsy, cerebral palsy, and multiple sclerosis. For studies evaluating developmental delays due to autism during childhood, select *Maternal/paternal/child/health* and *Neurological disease* as rationale. Note: *Alzheimer's disease* is listed separately because it is a leading cause of death. *Neurological disease* is a health condition, so it can be a rationale. It can be an exposure, for example, in a case-control study of whether Parkinson's disease patients are more likely to suffer from falls. It can be an outcome, for example, in a study of the development of neurological disorders such as early-onset dementia in football players with a history of traumatic brain injury. *Neurological disease* does not include cognitive decline; for these studies, select *Other or unclear*.

29. *Obesity*
Obesity is considered a disease, so obesity can be selected as a rationale. This topic includes overweight and studies of energy balance in the context of overweight and obesity. It includes measures of obesity, such as body mass index (BMI), adipose tissue, sustained weight loss, or waist circumference, in studies clearly studying overweight or obesity. It does not include physical activity studies or studies of energy balance unless the studies also focus on overweight/obesity. Studies that involve weight measures as exposures or outcomes that are outside the context of obesity or overweight should not be included in this topic (e.g., in a study examining BMI as a measure of wasting in seriously ill patients). If a study examines the relationship between BMI (as a measure of obesity) or body composition and subsequent disease, then obesity can be an exposure. If a study tests approaches to reduce obesity, then obesity can be an outcome.

30. *Physical activity*
Physical activity, including aerobic activity, resistance training, activity patterns, sedentary behavior, or inactivity, is the 2nd actual cause of death. It is not a health condition, so it cannot be a rationale. Studies of yoga, including those types of yoga that incorporate meditation, are included in *Physical activity*. For

© 2019 Vargas AJ et al. *JAMA Network Open.*

studies of meditation alone, select *Other or unclear*. Physical activity can be an exposure for example, in studies examining the relationship between physical activity or inactivity and subsequent disease. It can be an outcome, for example, in studies evaluating the effect of an intervention on subsequent physical activity levels. If a study includes measures of attitude, knowledge, beliefs, or intentions about physical activity or exercise, select *Other or unclear*. If a study is examining an educational intervention or therapy that engages participants in physical activity, select both *Education/counseling* and *Physical activity* as the exposure.

31. *Policy/built environment*
    This topic includes studies that examine the effects of existing policies established by laws/regulations or by organizations such as school policy, worksite policy, household policy, or healthcare policy. It also includes studies involving indoor/outdoor environmental structures in the community such as sidewalks, billboards, parks, tobacco and alcohol retail outlets, and other manmade environments. It also includes features of those environments such as land use, walkability and the availability of fruits and vegetables. This topic does not include studies of environmental health generally (e.g., studies examining the source or impact of environmental toxins). It is not a health condition, so it cannot be a rationale. It can be an exposure in a study examining the relationship between a policy or environment and a health condition. For example, if a study is evaluating how a higher sales tax on cigarettes influences smoking, it may be appropriate to select *Policy/built environment* as the exposure and *Tobacco* as the outcome. It can be an outcome if a study is examining approaches that could be used to change a health-related environment or policy. If the content of the policy focuses on another topic listed under A. Study Focus – e.g. *Alcohol*, *Substance abuse*, *Obesity*, *Tobacco* – it may be appropriate to select *Policy/built environment* and the other topic as the exposure and/or outcome. For example, for a study designed to change economic policy to influence tobacco control, select *Tobacco* as the outcome and *Policy/ built environment* as the exposure and outcome.

32. *Pneumonia/influenza*
    Pneumonia/influenza is the 9th leading cause of death. These conditions are listed together because death generally occurs from influenza if it progresses to pneumonia. This topic includes diagnostic measures of pneumonia/influenza, such as chest x-ray or plasma proteins. It is a health condition, so it can be a rationale. It can be an exposure, for example, in a study looking at whether previous exposure to pneumonia increases risk for chronic lung disease or cancer. It can be an outcome, for example, in studies examining interventions, medications, or other methods to prevent these infections. If the study is about pneumonia/influenza, select this topic instead of *Infectious disease*. This topic also includes testing and measures of pneumonia or influenza (e.g. viremia, antibodies, or viral blood count) indicating natural infection or immunity, not vaccine-induced immunity. For studies using measures of pneumonia or influenza that indicate vaccine-induced immunity, select *Vaccine* to represent these measures. If a study about pneumonia or influenza is examining drug-resistance or uses a similar term, select this topic because the drug-resistance is caused by changes in the infectious agent.

33. *Sexual behavior*
    Sexual behaviors are the 9th actual cause of death. This includes risky sexual behaviors like failure to use condoms outside of a stable monogamous relationship and sexual contact with people who have a sexually transmitted disease like HIV/AIDS or other sexually transmitted infections (STI). This topic includes other sexual behaviors which can also increase risk of disease under some circumstances. This topic includes measures of sexual behavior such as age at first sexual encounter or number of partners and the use of behavioral contraceptive methods such as condoms, abstinence, withdrawal method, or rhythm method. It does not include sexual identity. It is not a health condition, so it cannot be a rationale. It can be an exposure, for example, if a study is examining specific sexual behaviors increasing the risk for infectious disease like HIV or Hepatitis B or D. It can be an outcome, for example, in a study examining social or familial effects on sexual behaviors. If a study includes measures of attitude, knowledge, beliefs or intentions about sexual behaviors, select *Other or unclear*.

34. *Stress*
    Stress is not a health condition, so it cannot be a rationale. Stress is the body’s reaction to a stressor. Stressors can be anything (physical or psychological) that triggers a stress response in the body. Distress

© 2019 Vargas AJ et al. *JAMA Network Open.*

refers to stress induced by an unwelcome event or stress that exceeds the body's mechanisms for coping and is coded as *Stress*. Stress is of interest as a potential factor that influences health as an exposure (e.g., stress related to hypertension), or as an outcome (e.g., of an intervention study). This includes distress, psychosocial stress, stressors, but not resistance training (select *Physical Activity*), molecular/cellular stress (select *Other or unclear*), or PTSD (*Mental health*).

35. *Stroke*
    Stroke is the 4th leading cause of death, and includes hemorrhagic or ischemic stroke, cardiovascular events, and transient ischemic attacks (TIAs). It is a health condition, so it can be a rationale. It can be an exposure, for example, in a study that examines the risk of heart attacks in those who have already had a stroke. It can be an outcome, for example, in a study using physical activity interventions to prevent a second stroke. If a study cites cardiovascular disease (CVD) or peripheral artery disease (PAD), select both *Heart disease* and *Stroke*; but select only *Heart disease* if a study cites coronary heart disease (CHD). This topic does not include pre-clinical or subclinical cardiovascular disease; for these studies, select *Other or unclear* instead.

36. *Substance abuse*
    Illicit drug use is the 9th actual cause of death and is a diagnosable condition, so it can be a rationale. This includes use of illegal substances or abuse of prescription or legal substances (other than tobacco or alcohol) that could harm health. It also includes overdose and withdrawal from those substances and addiction to those substances. This topic includes measures of substance use/abuse such as age of onset or frequency of use as well as measures of substance abuse, such as drug-specific metabolites. It can be an exposure, for example, in a study examining the relationship of alcohol or substance abuse on developing liver disease. It can be an outcome, for example, in studies of interventions to prevent drug abuse. For studies about alcohol or tobacco use or addiction, select those topics instead of *Substance abuse*. For studies that examine addictive behavior disorders as an exposure or outcome, select *Substance abuse* and do not select *Mental Health*. This topic does not include the medical use of marijuana (select *Medication/device*). If a study includes measures of attitude, knowledge, beliefs, or intentions about substance abuse, select *Other or unclear*.

37. *Suicide*
    Suicide is the 10th leading cause of death, and includes suicidal ideation or intent as well as suicidal behavior (actual and attempted suicide by any means). It is a health condition, so it can be a rationale. It can be an exposure, for example, in a study that examines whether a prior suicide attempt leads to a previously undiagnosed mental disorder. It can be an outcome, for example, in studies testing suicide-prevention interventions or examining risk factors for suicide. If a study includes measures of attitude, knowledge, or beliefs, about suicide, select *Other or unclear*.

38. *Surgery*
    This topic includes any kind of surgery (e.g., by scalpel, laparoscope, radiosurgery, etc.) and the immediate pre-operative preparation for surgical procedures (e.g., using antibiotic wash on the patient, starting an intravenous line). Surgery is not a health condition, so it cannot be a rationale. It does not include long-term preparation for surgery which takes place outside of the surgical setting (e.g., taking prophylactic antibiotics, preparing organs for implant). Examples of surgery include circumcision, appendectomy, tonsillectomy, laparoscopy, biopsy, and skin grafting. This topic includes organ transplantation procedures, but does not include complications resulting from the transplant (e.g. graft versus host disease (GVHD), primary graft dysfunction (PGD), organ rejection); select *Other or unclear* for these conditions. Surgery can be an exposure (e.g., a study testing effects of bariatric surgery effects on risk of diabetes), but it also can be an outcome (e.g., a study is looking at factors that influence, or interventions to influence, the use of surgical procedures in practice).

39. *Tobacco*
    Tobacco use includes smoking, smokeless tobacco, e-cigarettes, other products derived from tobacco (e.g., lozenges, lollipops), tobacco addiction, smoking cessation, and regulatory science related to tobacco use. This topic includes self-reported measures of tobacco use such as age of onset or number of cigarettes smoked per day as well as objective measures of tobacco exposure through either secondhand smoke

© 2019 Vargas AJ et al. *JAMA Network Open.*

exposure or active smoking, such as cotinine or exhaled carbon monoxide. It can be a rationale because it is a diagnosable condition, and does influence numerous health conditions. Tobacco use can be an exposure (e.g., a study to find out whether tobacco use is a risk factor for cancer) or a study outcome (e.g., a study to encourage smokers to quit, where smoking status would be the outcome). If a study is examining the effect or impact of toxins from tobacco smoke, select *Chemical/toxin* as the exposure or outcome instead (in this case, it may be appropriate to select *Tobacco* as the rationale). This topic includes exposure to secondhand smoke from tobacco unless the study is measuring specific chemicals/toxins in which case, select *Chemical/toxin*. For a study designed to change economic policy to influence tobacco control, select *Tobacco* as the rationale and outcome and *Policy/built environment* as the exposure and outcome. For studies that examine nicotine dependence disorder as an exposure or outcome, select *Tobacco* and do not select *Mental health*.

Nicotine replacement products marketed with health claims should be coded under *Medication/device*, not under *Tobacco*. This would include nicotine replacement gum, nicotine patches, nicotine inhalers, nicotine nasal spray, or nicotine lozenges. Other products that deliver nicotine (e.g., e-cigarettes) cannot be marketed with health claims and so *Tobacco* should be selected for those products. If a study includes measures of attitude, knowledge, beliefs, or intentions about tobacco use, select *Other or unclear*. Note: tobacco plants may be used as a production platform for a drug that has nothing to do with nicotine or tobacco use as described here; in such cases, do not select *Tobacco* as a topic.

40. *Unintentional injuries*
Unintentional injuries are the 5th leading cause of death. This is a health condition, so it can be a rationale. This topic includes unintentional injuries or death from falls, fires, or unintentional drowning (if deaths are being studied, also select *Mortality*). It includes fractures, traumatic brain injuries, concussions, and spinal cord injuries. It can be an exposure, for example, in a study examining the risk of developing lung disease after exposure to smoke inhalation from fires. It can be an outcome, for example, in studies examining interventions or risk factors for falls in the elderly. This does not include injuries from motor vehicle crashes or firearms; for these studies, select *Motor vehicle crash* or *Firearms*, respectively.

41. *Vaccine*
Traditional vaccines contain either parts of microbes or whole microbes that have been killed or weakened so that they don't cause disease. When administered, the body develops antibodies so that if later confronted with the same microbe, the body is able to defeat it. Increasingly, the term vaccine is also used to describe drugs that induce the immune system to block the psychoactive and behavioral effects of nicotine and other addictive drugs. This topic is used broadly to include both traditional and newer types of vaccines. If *Vaccine* is selected, do not also select *Medication/device*.

Vaccine includes immunizations, vaccine delivery, studies of population-level immunity, adjuvants and other vaccine ingredients, and vaccine development. It also includes measures of immune response (e.g., antibodies) if those markers are used to indicate immune response to the vaccine. It is not a health condition, so it cannot be a rationale. It can be an exposure, for example, in studies testing the efficacy of a new vaccine. It can be an outcome, for example, in vaccine development studies or studies seeking to increase use of vaccines (e.g. vaccine initiation, vaccine-related behaviors). If a study includes measures of attitude, knowledge, beliefs, or intentions about vaccines, select *Other or unclear*. If, for example, a study is examining allergic reactions to the flu shot among people with an egg allergy, then it may be appropriate to select *Vaccine* as the exposure and *Other or unclear* as the outcome; do not select *Diet/nutrition*. This topic does not include vaccine escape phenotypes; because these studies are about viruses, select *Infectious disease*.

Some studies may examine cancer vaccines for prevention. Cancer prevention vaccines are similar to traditional vaccines because they protect against infectious agents, so it is appropriate to select *Vaccine* as exposure or outcome; they are not medications because they induce an immune response and do not treat cancer. For example, in a study examining the efficacy of a human papilloma virus (HPV) vaccine in protecting against incident cervical cancer, select *Infectious disease* (for HPV) and *Cancer* as the rationale, *Vaccine* as the exposure, *Cancer* as the outcome, and *Preventing a new health condition, promoting health, or identifying risk factors* as the F. Prevention research category. Cancer treatment vaccines are intended to

© 2019 Vargas AJ et al. *JAMA Network Open.*

treat an existing cancer, and it would be appropriate to select *Vaccine* as exposure or outcome, and *Other or unclear* under F. Prevention research category.

42. *Violence*
    Violence includes any threatening act or aggression such as assault, bullying, homicide (also select *Mortality*), sexual violence, elder abuse, and carrying or making threats (e.g., physical, verbal) with weapons other than firearms. It is not a health condition, so it cannot be a rationale. It can be an exposure, for example, in a study examining children who are bullied and their risk for developing mental health conditions as adults. This can be an outcome, for example, in violence prevention interventions. This does not include motor vehicle crashes or firearms; for these studies, select *Motor vehicle crash* or *Firearms*, respectively. *Violence* includes child maltreatment or child abuse only if physical abuse is specifically being studied; for these studies, also select *Maternal/paternal/child health* for rationale. If a study includes measures of attitude, knowledge, beliefs, or intentions about violence, select *Other or unclear*.

43. *Other or unclear*
    Select this topic as the rationale if the **health condition** serving as the rationale is not listed in A.1 Rationale. Do not select this topic as the rationale for a blacked out topic. Select this topic as the exposure or outcome when none of the topics listed apply (e.g., cost and cost-effectiveness). This topic should be selected for disease risk unless the risk is based on a measure of the health condition itself (e.g., incidence or prevalence). If a study is examining prevalence or incidence of a health condition, disease, or risk/protective factor, select *Other or unclear* as the exposure and the health condition, disease, or risk/protective factor as the outcome. If a study describes symptoms that are not diagnostic of a disease or condition (e.g. depressive symptoms, psychiatric symptoms, diabetes symptoms, coughing as a symptom), select *Other or unclear* as appropriate for exposure or outcome.

B. *[Removed]*

C. *[Removed]*

D. Population focus

The population groups listed below should be selected only if explicitly stated as being studied in the abstract and/or title (i.e., recruited, measures taken, interventions delivered). Other sources of information (other than the study abstract) will be available to provide information on race/ethnic and gender distributions, so they are not included in this list. More than one population may be selected if applicable; sometimes this information can be found when the abstract refers to recruitment sources. In a study with multiple population groups, select all that apply; if there is any group that does not fit, select *Other or unclear* to represent that group. If the abstract uses an indecipherable symbol followed by an age (e.g., "e60", "¿10"), then treat the symbol as equivalent to an "=" (equal sign). Special populations of interest are based on demographic characteristics, not disease status. They include the following:

1. *Incarcerated/institutionalized*
   This topic refers to residential populations in prisons, mental health institutions, rehabilitation facilities, nursing homes, and/or hospice care facilities. It includes participants that are recruited, measured, or given an intervention while they are incarcerated/institutionalized, even if they are not incarcerated/institutionalized for the duration of the study. It also includes participants that were released from incarceration/institutionalization if the focus of the study is on that population. Note: this topic focuses on those who are incarcerated/institutionalized long-term, so the terms "hospitalized patients" or "inpatients" are not sufficient to justify selecting *Incarcerated/institutionalized*.

2. *Sexual and gender minorities*
   This topic includes lesbian, gay, bisexual, transgender, intergender populations, and other sexual minorities. It also includes men who have sex with men (MSM).

3. *Low income*
   Use this topic when the investigator states "poor," "impoverished," "low wage," "low socioeconomic status," or similar term. This topic includes individuals who participate in assistance programs (e.g.,

© 2019 Vargas AJ et al. *JAMA Network Open.*

Medicaid, food stamps, WIC, SNAP, etc.) or are served by healthcare safety net providers (e.g., federally qualified health centers). This does not include child welfare, which is the equivalent of child protective services and distinct from financial welfare. Note: a disadvantaged population is not considered *Low income*, but an economically disadvantaged population (e.g., homeless) is considered *Low income*.

4. *Military/veterans*
   This topic includes all branches of the military and their reserves: Army, Air Force, Coast Guard, Marine Corps, and Navy, as well as the National Guard. The Commissioned Corps of the U.S. Public Health Service is a non-military uniformed service and therefore not included in this topic. This topic also includes military dependents as well as retired military and military veterans that are serviced by the medical facilities, clinics, and benefits offices of the U.S. Department of Veteran Affairs.

5. *Older adults/elderly*
   Use this topic when the investigator states "older adults" or the "elderly" and gives no specific ages, or when the age range may include age 65 and older. If the study also examines adults below age 65 then also select *Other or unclear* unless another topic is selected which describes the study population. This topic includes Medicare beneficiaries since a majority of this group is 65 and older.

6. *People with disabilities*
   Use this topic when the investigator uses the term "disability" or if the study includes people with an impairment that substantially limits one or more major life activities. This includes hearing impairments/deafness, vision impairments/blindness, developmental disabilities (e.g., autism spectrum disorder, Asperger's Syndrome), intellectual disabilities (e.g., Down syndrome, Prader-Willi syndrome, Fragile X syndrome, Angelman syndrome), learning disabilities (e.g., dyslexia, dyspraxia, dysgraphia), and physical disabilities (e.g., amputation, paralysis, cerebral palsy, spinal cord injury, multiple sclerosis). Note: not all people with mental illness are considered disabled; if the abstract gives context that those with mental illnesses have a disability, then it may be appropriate to select this topic.

7. *Pregnant and/or post-partum women*
   Use this topic when the investigator states "pregnant," "post-partum," or similar term that indicates pregnant women or women within one year of giving birth.

8. *Rural*
   Use this topic when the investigator states "rural," "agricultural," "village," or similar term.

9. *Urban*
   Use this topic when the investigator states "urban" or "inner city" or other similar term; or when a specific city is named. Note: when a study is a community-based study conducted in a city, select *Urban*.

10. *Youth*
    Use this topic for studies of fetuses, infants, children, or adolescents (<18 years). If the study also examines adults age 18 and older then also select *Other or unclear* unless another topic is selected which describes the study population.

11. *Other or unclear*
    Use this topic when a study population is not listed above or when there are no human subjects. For example, if the population is women only and in an urban setting, select *Urban* only, do not also select *Other or unclear*. Another example would be men who have sex with men; select only *Sexual and gender minorities* and not *Other or unclear*.

E. Study design/purpose

Below are descriptions of the most common study designs used in prevention research. Select all that apply for the particular abstract. More than one study design may be possible in an abstract. For example, an abstract could have a first phase that is an *Observational study* and a second phase that is a *Randomized intervention*

study. If *Analysis of existing data*, *Methods research*, or *Pilot/feasibility/proof-of-concept/safety study* are selected, it may be appropriate to select another topic to describe the study design.

An abstract describing a clinical trial to evaluate a medication, device, or treatment may use the following terminology referring to the phases of clinical trials:

- Pre-clinical: Research using animals to find out if a medication, device, procedure, or treatment is safe and likely to be useful. Pre-clinical studies take place before any testing in humans is done. Note: pre-clinical studies precede Phase I studies; for studies that are entirely pre-clinical studies, select only *Other or unclear* for the E. Study design/purpose and *Other or unclear* for the F. Prevention research category. However, if there is a second phase of the study, it may be appropriate to select another E. Study design/purpose. In those situations, do not code the pre-clinical part of the study.
- Phase I: Testing a new medication, device, or treatment in a small group of people to evaluate its safety and identify side effects. For these studies, select *Pilot/feasibility/proof-of-concept/safety*. Note: select *Other or unclear* under F. Prevention research category for Phase I clinical trials.
- Phase II: Testing a new medication, device, or treatment in a larger group of people to see if it is effective and further evaluate its safety. For these studies, select *Pilot/feasibility/proof-of-concept/safety*. Note: select *Other or unclear* under F. Prevention research category for Phase II clinical trials.
- Phase III: The medication, device, or treatment is given to large groups of people to confirm its effectiveness, compare it to commonly used treatments, and collect information that will allow the medication, device, or treatment to be used safely. If the study is designed as a randomized study, select *Randomized intervention study*. Otherwise, select *Non-randomized intervention study*.
- Phase IV: Studies done after the medication, device, or treatment has been marketed to gather information on the drug's effect in various populations and any side effects associated with long-term use. For these studies, select *Observational study.*

An abstract describing prevention research more generally may use the following terminology:

- Hypothesis development. These studies are often classified as *Analysis of existing data* or *Observational studies*
- Methods development. These studies are often classified as *Methods research*.
- Controlled intervention trials, also called efficacy trials, or explanatory trials. An intervention refers to any kind of exposure designed or intended to have a specific, non-transient effect on a health condition (disease, disorder, injury, disability) or risk/protective factor. Examples include provision of a new vaccine to prevent malaria infection, universal vs. targeted HIV screening in the emergency department, health education programs to encourage smoking cessation, and medication to treat high blood pressure. These studies are often classified as *Randomized intervention studies*, or if randomization was not used, as *Non-randomized intervention studies*.
- Defined population studies. These studies are often classified as *Randomized intervention studies*, or if randomization was not used, as *Non-randomized intervention studies*.
- Implementation projects, also called effectiveness trials, or pragmatic trials. These studies are often classified as *Randomized intervention studies*, or if randomization was not used, as *Non-randomized intervention studies*. They can also be *Observational studies*.

1. *Analysis of existing data*
   This topic is often called secondary data analysis. Select this topic when the study is analyzing data collected by others or collected in a previous study by the same investigators, such as for analysis of an existing dataset or of synthetic data, meta-analyses, simulation studies, development of risk prediction equations, or similar activities. If a study names an existing clinical trial, community trial, or epidemiologic study as a source of data, select *Analysis of existing data*. If the study is recruiting subjects or taking samples or biological specimens from the named trial or study, select *Analysis of existing data*. This topic

© 2019 Vargas AJ et al. *JAMA Network Open.*

includes studies that abstract information from existing records such as electronic health records, patient charts, claims records, etc. when the data are used in their existing form. The data need to exist only at the time the investigator accesses them. This topic does not include primary data collection. If the abstract proposes to use point estimates from existing data for validation, that is not considered part of this topic. For example, a study may use information about prevalence of a certain condition, gathered from an existing study, to validate a survey tool – this is not considered *Analysis of existing data.* Document-based research is not necessarily considered *Analysis of existing data*. For example, a study may analyze documents such as cigarette ads, press releases, or newspaper articles to identify and categorize common marketing messages used by the tobacco industry – this is not considered *Analysis of existing data* because the data must be manipulated in order to analyze it. Conversely, if a study uses patient charts or electronic health records to extract the weight and height of each patient in the sample, this study is considered *Analysis of existing data*. Selecting this topic does not preclude selecting other topics in E. Study design.

2. *Methods research*

   Select this topic for studies that are developing and/or validating new methods or improving existing methods. Examples include developing and/or evaluating different study designs, statistical analysis approaches, measurement approaches (including the development of new measures), recruitment strategies, survey instruments, screening methods, etc. This topic includes studies examining the sensitivity, specificity, and/or predictive value of a screening tool. It is not to be selected just because the study uses different or unusual methods, or adapts an existing method to a new population. It also does not include the development of interventions. Selecting this topic does not preclude selecting other topics in E. Study design.

   This topic does not include development or testing of basic research methods. It also does not include development or testing of diagnostic tools, unless they are explicitly used for screening or are explicitly described as screening tools. Studies that identify or discover biomarkers are not included in this topic; however, studies that develop and/or validate biomarkers are considered *Methods research*. If a study is developing a predictive model, it may be considered *Methods research;* however, using an existing predictive model is not considered *Methods research*.

   This topic includes simulation studies to compare different study design options or different statistical analysis approaches applied to prevention research questions; such studies generate new data and so are not examples of *Analysis of existing data*. It is not appropriate for all simulation studies, for example, where the study uses simulation methods to compare the effects of two possible policy choices on morbidity or mortality rates. Nor would it be appropriate for a molecular simulation study to develop a new medication.

3. *Non-randomized intervention study*

   Select this topic for studies examining causal effects of interventions but that do not randomize participants to receive, or not receive, the intervention. An intervention refers to any kind of exposure designed or intended to have a specific, non-transient effect on a health condition (disease, disorder, injury, disability) or risk/protective factor. If the terms “randomized,” “randomly assigned,” or “random assignment” are not used, select this topic for intervention studies where it is not clear how the study participants were assigned. These include the following type of studies:

   a. Quasi-experiments—intervention and control groups are determined by the investigators, but participants are not assigned randomly; includes pre-to-post or time series designs with a comparison group but no randomization.

   b. Pre-post study without an external control/comparison group—one measurement of study outcome occurs before and one occurs after an intervention is delivered; there is a within-subjects comparison.

   c. Multiple baseline or time series designs—multiple measurements of study outcomes occur before, during, and after an intervention is delivered; there may or may not be a comparison group.

   d. External comparison—a comparison group is not recruited or assigned by the investigators, but one outside the study is used. For example, county, state, or national data collected by others or historical data previously collected is used to form the comparison group.

e. Natural experiments where interventions are not delivered by the investigators, but are those that are being delivered by others or through policy or environmental changes, not under control of the investigator; outcomes in persons who receive the intervention are compared to those who don't receive it or receive less of it; also may be called outcomes research for such studies in clinical settings or with patients.

f. Other studies conducted to follow participants after completion of a non-randomized intervention study where the non-randomized design is maintained or used in the comparison.

g. If the study has an intervention but does not describe a design consistent with the six designs described above, select *Other or unclear*.

4. *Observational study*
Select this topic for studies where naturally occurring exposures and outcomes are measured and analyzed, and no intervention is delivered. An intervention refers to any kind of exposure designed or intended to have a specific, non-transient effect on a health condition (disease, disorder, injury, disability) or risk/protective factor. This topic includes the following study designs:

a. Case-control study—starts with cases who have the condition/disease, identifies controls (often by matching on age, gender, etc.), and compares prior exposures to predict the condition/disease.

b. Cohort study—starts with people who do not have the condition/disease, measures exposures, and follows the people over time to identify onset of the condition/disease or health outcomes in those exposed compared to not exposed.

c. Cross-sectional study—exposures and outcomes are measured at the same time; includes prevalence studies and population surveys.

d. Ecological study—observational study where the unit of analysis is groups of people or geographic areas such as counties, states, or nations.

e. Other observational study designs, such as case-control studies nested in a cohort study, observational studies conducted to follow participants after completion of a randomized controlled trial where the randomized design is no longer maintained or used in the comparison, or other designs.

f. This topic includes genome-wide association studies (GWAS).

5. *Pilot/feasibility/proof-of-concept/safety study*
Select this topic when the study description uses "pilot," "feasibility," "proof-of-concept," "safety," or similar terms (e.g., acceptability, usability) in the context of testing a procedure. The study could be in animals or humans, observational or intervention. This topic includes studies that are testing whether an intervention is culturally acceptable to a population, but does not include studies that are only using a "culturally acceptable" intervention. Studies testing the effectiveness or efficacy of an intervention are not included in this topic. Note: side effects of medications/devices may be used to evaluate safety and/or toxicity; it may be appropriate to select *Medication/device* under A. Study Focus and *Pilot/feasibility/proof-of-concept/safety*. Selecting this topic does not preclude selecting other topics in E. Study design.

6. *Randomized intervention study*
Look for the terms "randomized," "randomly assigned," or "random assignment." Note: this is not the same as random selection/sampling or a randomization test.

Randomized controlled trials where the intervention is delivered by investigators; includes the following study designs:

a. Individually randomized controlled trials (RCT)—individual subjects are randomly assigned to intervention or control group

© 2019 Vargas AJ et al. *JAMA Network Open.*

b. Group randomized trials (GRT)—groups of people, organizational entities (such as schools or worksites), or entire communities are randomly assigned to intervention or control group; also called cluster randomized trials.

c. Other randomized designs, such as factorial randomized trials, stepped wedge trials, studies conducted to follow participants after completion of a randomized controlled trial where the randomized design is maintained, or other designs.

d. This topic does not include simulation studies, even if they used randomization.

7. *Other or unclear*

Select this topic if the design/purpose is not listed in one of the categories above or if you cannot tell from the abstract whether the study fits into one of the above options. For example, select this topic for studies where an intervention is being delivered, no randomization, and no indication of a comparison group. For basic research or pre-clinical studies, select *Other or unclear*. Basic research whose purpose is an understanding of biological structure, biological mechanisms, or behavioral mechanisms should be coded *Other or unclear*.

F. Prevention research category

This category groups studies into topics of prevention research that should enable persons interested in subtypes of prevention research to identify studies within that subtype, and to allow for consideration of what is considered prevention. Therefore, the topics are designed to accommodate various definitions of prevention.

Think about the research as described in the abstract and select one or more of the following categories of prevention. If you are not sure, please select *Other or unclear*.

1. *Preventing a new health condition, promoting health in the general population, or identifying risk factors for a new health condition*

a. This topic is often called primary prevention or health promotion.

b. Factors of interest include health behaviors, environmental exposures, and biological risk or protective factors (including biomarkers) that may increase or reduce risk of a new health condition or promote health.

c. Includes observational studies that identify risk and/or protective factors for onset of a new health condition or for promoting optimal health. Includes risk prediction studies including genetic risk and other factors that may increase risk of a health condition.

i. This topic does not include identification of risk factors for disease recurrence; for those studies, select *Preventing progression of disease, preventing recurrence in those with a known health condition, identifying risk factors for progression or recurrence.*

d. Includes development and evaluation of interventions to reduce risk of a new health condition, such as improving health behaviors and decreasing identified risk factors.

e. Includes intervention studies that are testing prevention or health-promotion interventions in apparently healthy people without an existing health condition.

f. Includes preventing a new health condition in people who already have a health condition that may increase risk of the new condition.

g. Examples include: promoting physical activity and healthy diet in the general population; preventing smoking initiation; preventing first heart attacks; preventing new primary cancer after remission for a different cancer; tobacco control policies to prevent smoking initiation; preventing high blood pressure; preventing injurious motor vehicle crashes; preventing injuries from falls; preventing CVD in cancer survivors; preventing the progression of precancerous conditions/lesions to cancer; prevention of the development of Barrett's esophagus, as well as the development of esophageal cancer in patients with Barrett's esophagus; preventing suicide in people with depression; promoting healthy diet and

© 2019 Vargas AJ et al. *JAMA Network Open.*

exercise in people with diabetes to prevent CVD; provision of metformin or diet/nutrition interventions to prevent pre-diabetes from developing into diabetes; evaluating vaccines to prevent infectious disease in humans through an efficacy trial; mastectomy to prevent breast cancer in someone who has never had it before but is at high risk; ART treatment in people with HIV to prevent transmission of HIV; preventing incidence or transmission of drug resistant infectious disease.

2. *Screening for risk factor*
    a. Screening is defined as testing for known risk or protective factors in people not previously known to have a certain health condition to identify individuals or groups at risk for that health condition.
    b. This topic is considered part of primary prevention.
    c. Includes screening to detect asymptomatic and unidentified risk factors, and/or asymptomatic risk conditions. The study may be looking at effects or outcomes from an existing screening test. However, not all studies involving screening for a disease mean that the study is about screening. For example, a study may be screening patients for eligibility and then assigning eligible patients to an intervention. In contrast, if participants are assigned to an SBIRT intervention where screening is part of the intervention, then select this topic or *Screening for early disease* depending on the purpose of the screening.
    d. Excludes methods research that is developing a screening tool or method (select *Methods research*) unless the screening tool or method is being applied after it has been validated. Also excludes basic research studies examining biological mechanisms that may ultimately lead to a screening test, because they do not have immediate relevance for primary or secondary prevention research (select *Other or unclear*). Also excludes basic research that may use the term "screening," such as high throughput screening (HTS) of molecules for drug development.
    e. Includes studies establishing the prevalence of risk or protective factors in a population or subpopulation.
    f. Examples include: detection of elevated blood pressure by routine BP measurement (a risk factor for stroke), screening for physical inactivity or poor diet in people without a diagnosis, and screening for cervical dysplasia by Pap smear.

3. *Screening for early disease*
    a. Screening for early disease is defined as testing people not previously known to have a certain health condition to identify individuals or groups who have early, pre-clinical disease, for which they have not sought care and are asymptomatic. However, not all studies involving screening for a disease mean that the study is about screening. For example, a study may be screening patients for eligibility and then assigning eligible patients to an intervention. In contrast, if participants are assigned to an SBIRT intervention where screening is part of the intervention, then select this topic or *Screening for risk factor* depending on the purpose of the screening. If the screening is not explicitly for early disease, then select *Screening for risk factor*.
    b. This topic is considered part of secondary prevention. Note: if the study subjects have sought care for symptoms or a condition, then it is not screening, it is diagnostic testing or evaluation.
    c. Excludes methods research that is developing a screening tool or method (select *Methods research*) unless the screening tool or method is being applied after it has been validated. Also excludes basic research studies examining biological mechanisms that may ultimately lead to a screening test, because they do not have immediate relevance for primary or secondary prevention research (select *Other or unclear*). Also excludes basic research that may use the term "screening," such as high throughput screening (HTS) of molecules for drug development.
    d. Examples include: detection of breast cancer by routine mammography; detection of colon cancer by routine colonoscopy; and detection of HIV infection through screening of high-risk persons.

© 2019 Vargas AJ et al. *JAMA Network Open.*

e. Includes studies establishing the incidence or prevalence of early disease in a population or subpopulation.

4. *Preventing progression of disease, preventing recurrence in those with a known health condition, identifying risk factors for progression or recurrence*

   a. Identification of, or intervention for, behaviors and risk/protective factors (including biomarkers) that affect risk for progression of disease or recurrence of a known health condition.

   b. Includes observational studies to identify health behaviors or other potential risk or protective factors for progression or recurrence as well as intervention studies to evaluate interventions to improve health behaviors or other risk/protective factors in order to prevent progression or recurrence. This also includes screening to identify progression or recurrence or risk/protective factors for progression or recurrence.

   c. Some people consider this primary prevention, whereas others consider it secondary prevention.

   d. This topic does not include treatment or management of a diagnosed health condition unless that treatment is being evaluated in that study for its effect on progression or recurrence. There are five exceptions: treatment for smoking, drug abuse, alcohol abuse, weight loss treatment for overweight/obese populations, and preventing those with suicidal ideation or behaviors from committing suicide. For studies that involve other treatment situations or chronic conditions with predictable episodic recurrence (e.g. mental health, asthma, diabetes), select *Other or unclear.* Preventing the progression of any disease to death is considered treatment and should be coded as *Other or unclear*.

   e. Examples include: prevention of spontaneous mutation from non-resistant infectious disease to drug resistant infectious disease; promotion of heart-healthy nutrition in subjects who have had a heart attack to reduce risk of another heart attack; promotion of exercise to reduce risk of a recurrent cancer; exercise rehabilitation to reduce risk of another heart attack; treatment of high blood pressure to reduce risk of another stroke; antiretroviral therapy (ART) to prevent progression from HIV to AIDS; prevention of diabetic retinopathy; prevention of development of AIDS-related malignancies; tobacco control policies for smoking cessation; behavioral interventions to prevent relapse for alcoholism or substance abuse; preventing smoking relapse; aspirin to prevent another stroke in subjects who already had a stroke; adjuvant treatment using tamoxifen or radiation to prevent recurrence of breast cancer after breast cancer treatment; mastectomy to prevent another breast cancer; behavioral interventions to reduce risk of reinfection of an infectious disease that has been previously treated; promoting weight loss in overweight/obese populations; repeat bone scans to monitor progression of osteoporosis, either as a form of screening or to evaluate an intervention to prevent progression; repeat carotid ultrasounds to monitor progression of atherosclerosis, either as a form of screening or to evaluate an intervention to prevent progression; preventing those with suicidal ideation or behaviors from committing suicide.

5. *Methods research*

   Select this topic for studies that are developing and/or validating new methods or improving existing methods. Examples include developing and/or evaluating different study designs, statistical analysis approaches, measurement approaches, recruitment strategies, survey instruments, screening methods, etc. This topic includes developing and validating predictive models, which should not be confused with animal models or theoretical or logic models and frameworks. This topic includes studies examining the sensitivity, specificity, and/or predictive value of a screening tool. It is not to be selected just because the study uses different or unusual methods, or adapts an existing method to a new population. It also does not include development of interventions.

   Select only *Methods research* if the investigator is developing and/or validating a method, but does not apply it beyond the validation stage. If the method is applied beyond the validation stage (e.g., to identify a risk/protective factor, prevent a specific disease or condition, or screen for a risk/protective factor or early disease), then it may be appropriate to select another topic in F. Prevention research category. For example,

© 2019 Vargas AJ et al. *JAMA Network Open.*

if a study is developing a predictive model, it may be considered *Methods research*; however, using an existing predictive model is not considered *Methods research*.

This topic does not include development or testing of basic research methods. It also does not include development or testing of diagnostic tools, unless they are explicitly used for screening or explicitly described as screening tools.

Studies that identify or discover biomarkers are not included in this topic; however, studies that develop and/or validate biomarkers are considered *Methods research*. For studies that identify or discover biomarkers that mark onset of disease or disease progression/recurrence, select *Preventing a new health condition, promoting health in the general population, or identifying risk factors for a new health condition* or *Preventing progression of disease, preventing recurrence in those with a known health condition, identifying risk factors for progression or recurrence* as appropriate. For studies of biomarkers for treatment response or prognosis as it relates to treatment, select *Other or unclear*.

This topic includes simulation studies to compare different study design options or different statistical analysis approaches applied to prevention research questions. It is not appropriate for all simulation studies, for example, where the study uses simulation methods to compare the effects of two possible policy choices on morbidity or mortality rates. Nor would it be appropriate for a molecular simulation study to develop a new medication.

Methods studies must have immediate relevance for prevention research (i.e. the results could be applied in new prevention studies without further development) and not be an early step that will eventually lead to application in prevention research.

6. *Other or unclear*

Select this topic when you cannot reasonably place the study into a topic above.

If any of topics 1-5 in F. Prevention research category are selected, do not select *Other or unclear*. Basic research whose purpose is an understanding of biological structure, biological mechanisms, or behavioral mechanisms should be coded *Other or unclear* because it is too distal to be considered primary or secondary prevention research. Just as biological mechanisms are those biological processes that underpin biological phenomena, behavioral mechanisms are the processes that explain or underlie observed behavioral phenomena. Behavioral mechanisms could include but are not limited to: executive control, emotion regulation, metacognition, interoception, social regulation of behavior, and decision-related processes, such as valuation, risk perception, temporal discounting and social influence.

Treatment, including management of chronic conditions such as asthma, HIV/AIDS, and diabetes; studies investigating sequelae or complications of treatment; treatment to prevent progression of disease to death; rehabilitation studies; studies about palliative care; and studies developing new medications or therapies for treatment and rehabilitation should be coded *Other or unclear*. There are five exceptions: studies investigating sequelae or complications of treatment for smoking, drug abuse, and alcohol abuse, weight loss treatment for overweight/obese populations, and preventing those with suicidal ideation or behaviors from committing suicide. These should be coded *Preventing progression of disease, preventing recurrence in those with a known health condition, identifying risk factors for progression or recurrence*. For studies developing medications, vaccines, or devices that may eventually be used in prevention research, select *Other or unclear*. For pre-clinical studies as well as Phase I and Phase II clinical trials, select *Other or unclear*.

© 2019 Vargas AJ et al. *JAMA Network Open.*

Abstract Coding Form

Rater: ____________________
Date: ____________________

Prevention Taxonomy Form
CHECK ALL THAT APPLY IN EACH COLUMN
(TOPICS ARE NOT MUTUALLY EXCLUSIVE)
See accompanying protocol for definitions and examples

Appl ID_________ PI Last Name: _______________ Project Title: ___________________________________

**Study focus**	Rationale	Exposure	Outcome
1. Alcohol			
2. Alzheimer's disease			
3.			
4. Blood disorder			
5. Blood pressure			
6. Cancer			
7. Chemical/toxin			
8. Cholesterol			
9. Diabetes			
10. Diet/nutrition			
11. Education/counseling			
12. Firearms			
13. Gastrointestinal disease			
14. Genetics			
15. Healthcare delivery			
16. Heart disease			
17. HR quality of life			
18. Infectious disease			
19. Kidney disease			
20. Lung disease			
21. Maternal/paternal/child health			
22. Medication/device			
23. Mental health			
24. Microbiome			
25. Mortality			
26. Motor vehicle crash			
27. Musculoskeletal disease			
28. Neurological disease (not Alzheimer's)			
29. Obesity			
30. Physical activity			
31. Policy/built environment			
32. Pneumonia/influenza			
33. Sexual behavior			
34. Stress			
35. Stroke			
36. Substance abuse			
37. Suicide			
38. Surgery			
39. Tobacco			
40. Unintentional injuries			
41. Vaccine			
42. Violence			
43. Other or unclear			

**Population focus**	
1. Incarcerated/institutionalized	
2. LGBTI	
3. Low income	
4. Military/veterans	
5. Older adults/elderly	
6. People with disabilities	
7. Pregnant and/or post-partum women	
8. Rural	
9. Urban	
10. Youth (infants, children, adolescents)	
11. Other or unclear	

**Study design/purpose**	
1. Analysis of existing data	
2. Methods research	
3. Non-randomized intervention study	
4. Observational study	
5. Pilot/feasibility/proof-of-concept/safety	
6. Randomized intervention study	
7. Other or unclear	

**Prevention research category**	
1. Preventing new health condition, promoting health in the general population, or identifying risk factors for a new health condition	
2. Screening for risk factor	
3. Screening for early disease	
4. Preventing progression of disease, preventing recurrence in those with a known health condition, identifying risk factors for progression or recurrence	
5. Methods research	
6. Other or unclear	

© 2019 Vargas AJ et al. *JAMA Network Open.*

Appendix for Coding Complex Grants

Complex grants (e.g., U54, P01, P50, U19) include a parent award along with many subprojects and possibly 1-2 cores. When coding one of these complex grants, there is an overall “parent” abstract followed by a “subproject” abstract, with a line separating the two. Use the title, parent abstract, subproject abstract, and public health relevance to code A.1 Rationale. Generally, the title, subproject abstract, and public health relevance should be used to code the other categories. In some cases, the parent abstract might provide details specific to the subproject being coded. For example, if the parent abstract mentions that Project 2 will study patients in Los Angeles, but the subproject (which explicitly describes Project 2) doesn’t mention where the study is being conducted, then it is appropriate to select D.9 *Urban*.

If it is unclear what the investigator is proposing in the subproject, code A.1 Rationale and select *Other or unclear* for the other categories. If a subproject is only training research staff or supporting infrastructure (e.g., biobank, administration core, computing support, data management/support), select the health condition motivating the research for A.1 Rationale, and select *Other or unclear* for the other categories since we do not code training aims or infrastructure aims and the subproject is not conducting prevention research. If a subproject is only developing infrastructure for a basic science project, select only E.7 *Other or unclear* and F.6 *Other or unclear*. On the other hand, if a subproject has both infrastructure aims and prevention research aims (i.e., it is a partial), then code only the prevention research aims.

Some subprojects within these complex mechanisms may propose to fund pilot studies. For those subprojects, select *Pilot/feasibility/proof-of-concept/safety study* under E. Study design/purpose. However, if a subproject abstract aim indicates that researchers will be trained to perform a pilot research study, do not code that aim.

Subprojects may also include aims that propose training investigators to obtain new skills. These aims should not be coded as *Education/counseling* as this is an infrastructure activity.

eTable 1. Prevention Research Where No Leading Risk Factor or Cause of Death Was Measured as an Exposure or Outcome by Study Exposure or Outcome

**Study exposure and/or outcome**	**NIH prevention research portfolio, % (95% CI)**
Other/unclear	86.0% (83.4%-88.4%)
Genetics	32.0% (28.8%-35.4%)
Infectious disease[a]	16.7% (14.4%-19.2%)
Education/counseling	12.3% (10.9%-13.9%)
Medication/device	11.6% (9.4%-14.1%)
Mental health	10.7% (8.9%-12.7%)
Healthcare delivery	10.0% (8.5%-11.6%)
Neurological disease[b]	9.5% (7.6%-11.9%)
HRQOL	5.6% (4.5%-7.0%)
Stress	4.0% (3.1%-5.2%)
Vaccine	3.1% (2.0%-4.8%)
Microbiome	3.0% (2.0%-4.4%)
Gastrointestinal disease	3.0% (1.8%-4.9%)
Sexual behavior	2.8% (2.3%-3.4%)
Chemical/toxin[c]	2.6% (1.9%-3.5%)
Violence	2.6% (1.9%-3.5%)
Musculoskeletal disease	2.5% (1.6%-3.7%)
Policy/built environment	2.3% (1.8%-2.8%)
Mortality	2.0% (1.6%-2.5%)
Kidney disease[d]	1.7% (1.0%-2.9%)
Lung disease[e]	1.4% (0.8%-2.3%)
Heart disease[f]	1.2% (0.9%-1.6%)
Surgery	1.2% (0.6%-2.2%)
Stroke[g]	0.6% (0.4%-0.9%)
Blood disorder	0.5% (0.3%-0.8%)
Firearms	0.2% (0.1%-0.3%)[h]

[a] This category does not include pneumonia/influenza. There is a separate category below for pneumonia/influenza.
[b] This category does not include Alzheimer's disease. There is a separate category below for Alzheimer's disease.
[c] This category includes chemical/toxins beyond air pollution and beyond the Global Burden of Disease definition of air pollution.
[d] This category includes congenital kidney defects and urinary tract infections which are not in the CDC definition of Kidney disease.
[e] This category includes cystic fibrosis, pulmonary fibrosis, lung injuries, and pulmonary hypertension which are not the CDC definition of chronic lower respiratory disease.
[f] This category includes congenital heart disease which is not in the CDC definition of heart disease.
[g] This category includes Venous Thromboembolic Disease and unspecified stroke risk factors which are not in the CDC definition of stroke.
[h] Fewer than 10 research projects were manually coded in this category which may make estimates from these data unstable

© 2019 Vargas AJ et al. *JAMA Network Open.*

eTable 2. Prevention Research Where Leading Causes of Death Were Measured as an Exposure or as an Outcome

**Leading Causes of Death[a]**	**Exposure % (95% CI)**	**Outcome % (95% CI)**	**CDC,[a] % attributable deaths**
Any Top 10 Leading Cause of Death	2.0% (1.7%-2.4%)	25.2% (23.3%-27.1%)	74.0%
1) Heart disease	0.1% (0.1%-0.2%)	4.1% (3.3%-5.2%)	23.0%
2) Cancer	0.4% (0.3%-0.5%)	11.8% (10.4%-13.3%)	21.3%
3) Accidents	0.2% (0.1%-0.4%)	1.7% (1.3%-2.1%)	5.7%
4) Chronic lower respiratory disease	0.1% (0.0%-0.2%)[b]	1.6% (1.1%-2.4%)	6.0%
5) Stroke	0.1% (0.0%-0.2%)[b]	2.7% (2.1%-3.4%)	5.2%
6) Alzheimer's disease	0.0% (0.0%-0.1%)[b]	2.0% (1.4%-2.6%)	4.3%
7) Diabetes	0.8% (0.6%-1.0%)	3.1% (2.6%-3.8%)	3.0%
8) Influenza/Pneumonia	0.1% (0.0%-0.2%)[b]	0.5% (0.2%-1.0%)	2.0%
9) Kidney disease	0.3% (0.2%-0.6%)	1.3% (0.8%-2.0%)	1.8%
10) Suicide	0.0% (0.0%-0.1%)[b]	0.7% (0.5%-0.9%)	1.7%

[a] The top 10 leading causes of death in the U.S. for 2017 from the Centers for Disease Control (CDC) report Mortality in the United States, 2017. https://www.cdc.gov/nchs/data/databriefs/db328_tables-508.pdf#4. Accessed April 4th, 2019.

[b] Fewer than 10 research projects were manually coded in this category which may make estimates from these data unstable

© 2019 Vargas AJ et al. *JAMA Network Open.*

eTable 3. Prevention Research Where Leading Risk Factors for Death Were Measured as an Exposure or as an Outcome

**Leading Risk Factors for Death[a]**	**Exposure % (95% CI)**	**Outcome % (95% CI)**	**GBD,[a] % attributable deaths**
Any Top 10 Risk Factor for Death	15.0% (13.8%-16.2%)	26.3% (24.7%-28.0%)	57.3%
1) Dietary risk	5.2% (4.5%-6.0%)	3.5% (3.0%-4.0%)	19.1%
2) Tobacco	2.6% (2.1%-3.4%)	5.1% (4.4%-5.9%)	17.8%
3) High systolic blood pressure	0.6% (0.4%-0.7%)	2.3% (1.8%-2.9%)	17.4%
4) High body mass index	1.3% (1.0%-1.7%)	4.5% (4.0%-5.1%)	13.9%
5) High fasting plasma glucose	1.0% (0.7%-1.5%)	3.9% (3.2%-4.6%)	13.6%
6) High total cholesterol	0.5% (0.4%-0.7%)	1.4% (1.1%-1.9%)	8.4%
7) Impaired kidney function	0.4% (0.2%-0.6%)	1.4% (0.9%-2.1%)	6.3%
8) Alcohol/drug use	3.5% (3.0%-4.0%)	9.6% (8.6%-10.6%)	5.6%
9) Air pollution	1.2% (1.0%-1.4%)	0.4% (0.3%-0.5%)	3.8%
10) Low physical activity	2.4% (2.1%-2.8%)	3.3% (2.8%-4.0%)	3.3%

[a] The top 10 leading risk factors for death in the U.S. for 2016 from the Global Burden of Disease (GBD) recent publication: U. S. Burden of Disease Collaborators, Mokdad AH, Ballestros K, et al. The State of US Health, 1990-2016: Burden of Diseases, Injuries, and Risk Factors Among US States. *JAMA.* 2018;319(14):1444-1472.

© 2019 Vargas AJ et al. *JAMA Network Open.*

eFigure 1. Trends in Prevention Research That Measured a Leading Cause of Death as an Exposure or Outcome

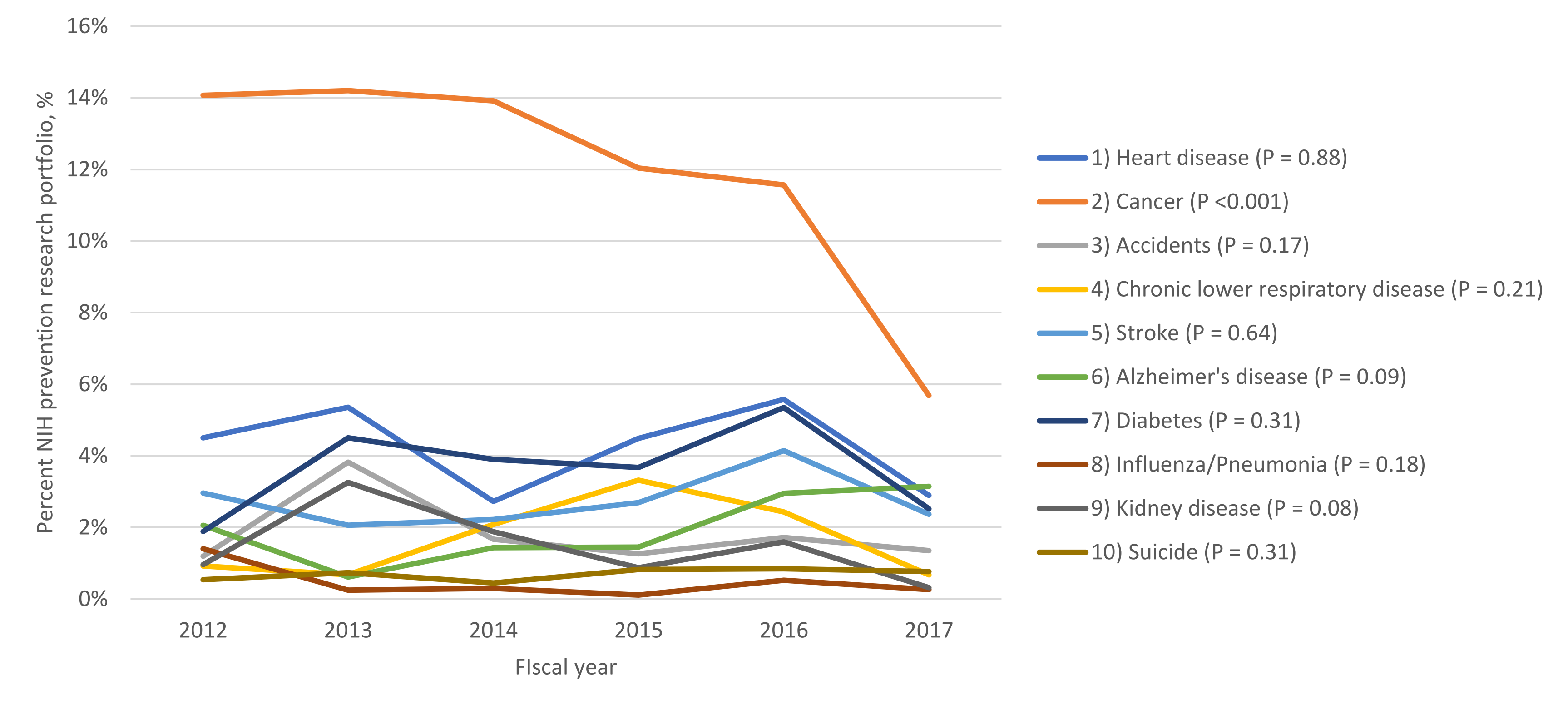

The percent of prevention research focused on any one of the top 10 leading causes of death in the U.S. was stable between fiscal years 2012-2017 with the exception of cancer. There was a large decrease in the amount of cancer prevention research in 2017 (P-trend = <0.001) due to a decrease in the amount of prevention research supported by the National Cancer Institutes and by other institutes. If any aim of a prevention research project was measuring a leading risk factor or cause of death as an exposure or outcome for a hypothesis, then the prevention research project was considered to focus on those leading risk factors or causes.

© 2019 Vargas AJ et al. *JAMA Network Open.*

eFigure 2. Trends in Prevention Research That Measured a Leading Risk Factor for Death as an Exposure or Outcome

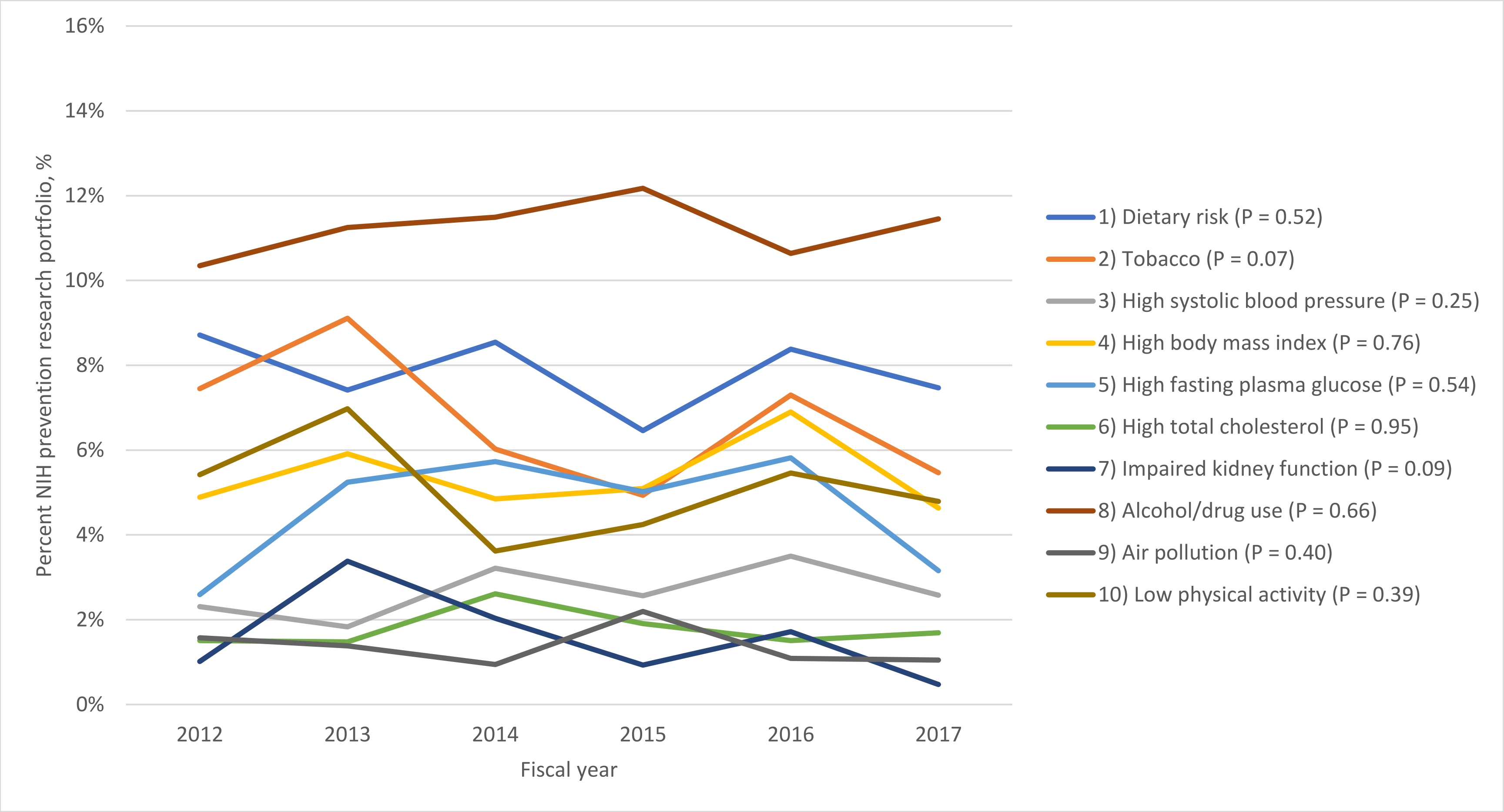

The percent of prevention research focused on any of the top 10 leading risk factors for death in the U.S. was stable between fiscal years 2012-2017 (all P-trend >0.05). If any aim of a prevention research project was measuring a leading risk factor or cause of death as an exposure or outcome for a hypothesis, then the prevention research project was considered to focus on those leading risk factors or causes.

© 2019 Vargas AJ et al. *JAMA Network Open.*
